# Untargeted metagenomic sequencing identifies Toscana virus in patients with idiopathic meningitis, southern Spain, 2015 to 2019

**DOI:** 10.2807/1560-7917.ES.2023.28.45.2200913

**Published:** 2023-11-09

**Authors:** Fabiana Gámbaro, Ana Belén Pérez, Matthieu Prot, Eduardo Agüera, Artem Baidaliuk, María Paz Sánchez-Seco, Luis Martínez-Martínez, Ana Vázquez, María Dolores Fernandez-Garcia, Etienne Simon-Loriere

**Affiliations:** 1 G5 Evolutionary Genomics of RNA Viruses, Institut Pasteur, Université Paris Cité, Paris, France; 2 Hospital Universitario Reina Sofía, Córdoba, Spain; 3 Instituto Maimónides de Investigación Biomédica de Córdoba (IMIBIC), Córdoba, Spain; 4 CIBER de Enfermedades Infecciosas (CIBERINFEC), Instituto de Salud Carlos III, Madrid, Spain; 5 Universidad de Córdoba, Córdoba, Spain; 6 National Centre for Microbiology, Instituto de Salud Carlos III, Madrid, Spain; 7 CIBER de Epidemiología y Salud Pública (CIBERESP), Instituto de Salud Carlos III (ISCIII), Madrid, Spain; 8 These authors contributed equally to this work and share last authorship and correspondence

**Keywords:** Untargeted metagenomic sequencing, Amplicon-based sequencing, Toscana virus, reassortment, viral meningitis

## Abstract

**Background:**

Various pathogens, including bacteria, fungi, parasites, and viruses can lead to meningitis. Among viruses causing meningitis, Toscana virus (TOSV), a phlebovirus, is transmitted through sandfly bites. TOSV infection may be suspected if patients with enterovirus- and herpesvirus-negative aseptic (non-bacterial) meningitis recall recent insect bites. Other epidemiological factors (season, rural area) may be considered. The broad range of possible meningitis aetiologies poses considerable diagnosis challenges. Untargeted metagenomic next-generation sequencing (mNGS) can potentially identify pathogens, which are not considered or detected in routine diagnostic panels.

**Aim:**

In this retrospective, single-centre observational study, we investigated mNGS usefulness to understand the cause of meningitis when conventional approaches fail.

**Methods:**

Cerebrospinal fluid (CSF) samples from patients hospitalised in southern Spain in 2015–2019 with aseptic meningitis and no aetiology found by conventional testing, were subjected to mNGS. Patients’ demographic characteristics had been recorded and physicians had asked them about recent insect bites. Obtained viral genome sequences were phylogenetically analysed.

**Results:**

Among 23 idiopathic cases, TOSV was identified in eight (all male; median age:  39 years, range: 15–78 years). Five cases lived in an urban setting, three occurred in autumn and only one recalled insect bites. Phylogenetic analysis of TOSV segment sequences supported one intra-genotype reassortment event.

**Conclusions:**

Our study highlights the usefulness of mNGS for identifying viral pathogens directly in CSF. In southern Spain, TOSV should be considered regardless of recalling of insect bites or other epidemiological criteria. Detection of a disease-associated reassortant TOSV emphasises the importance of monitoring the spread and evolution of phleboviruses in Mediterranean countries.

**Key public health message**

**What did you want to address in this study?**

In a high proportion of meningitis cases, the causative pathogen remains unknown despite extensive diagnostic testing. Unlike conventional testing, which targets known or suspected pathogens, metagenomic next-generation sequencing (mNGS) can potentially capture genomic data from any pathogen in a patient sample. We investigated by mNGS samples of patients hospitalised in southern Spain in 2015–2019 with undetermined cause of aseptic meningitis.

**What have we learnt from this study?**

Toscana virus (TOSV), a virus transmitted by sandflies, was identified in eight of 23 samples from patients with meningitis of unknown origin. Of the eight TOSV-positive patients, three had occurred in autumn and five lived in urban areas. Only one recalled recent insect bites, an event that may raise suspicion of TOSV infection. By analysing the genetic information of TOSV strains affecting the patients, insights into how TOSV evolves and spreads were gained.

**What are the implications of your findings for public health?**

The study reveals the utility of mNGS and the need to consider including TOSV in the differential diagnosis of aseptic meningitis in southern Spain regardless of the recall of insect bites or environmental conditions such as summer-season or rural residence. Panels for laboratory routine diagnostic of neurological infections in this region’s hospitals might also target TOSV, since timely diagnosis is crucial for public health risk management strategies.

## Introduction

Neurological infections are a considerable cause of morbidity and mortality worldwide, with 10.6 million viral or bacterial meningitis cases reported for 2017 alone [1]. Various pathogens, including viruses, bacteria, fungi, and parasites, can lead to meningitis and encephalitis [2]. Since the mid-2000s, molecular diagnostic tests have improved rapidly and multiplex PCR approaches now allow with high sensitivity and specificity to detect a wide variety of pathogens that can occur in cerebrospinal fluid (CSF). Despite the high global prevalence of central nervous system (CNS) infections and improved differential diagnostic methods, an investigation in the United Kingdom in 2011–2014 found that the reason for meningitis remained unknown in a large proportion (42%) of cases [3]. Moreover, a study in Houston, Texas, between 2005 and 2010, estimated that for 81% of patients with meningitis the cause was undetermined [4].

Diagnosing meningitis can indeed entail challenges. One of these is the broad range of potential aetiological agents, which cannot be singled out or deduced from the clinical presentation of patients alone. For finding the cause, several selective tests may be required, potentially raising considerations on their availability and costs. Another challenge is that pathogen-specific diagnostic assays (molecular or antigenic) may fail to detect a given pathogen because of genetic divergence. Furthermore, diagnostic tests may not be designed to detect pathogens, which are not suspected or commonly known to cause meningitis, such as emerging or re-emerging ones that have newly acquired this ability. An example of a virus for which the clinical picture resulting from infection appeared to change is Zika virus, which despite being known since 1947, was only associated with a risk of neurological afflictions in 2014–2016, upon its extensive spread in the Pacific and South America [5]. Last, as the amount of pathogen in CSF may vary from the moment of infection onwards, the timing of a patient seeking medical care or the timing of sampling may influence the chance of reliable detection (due to clearance of the pathogen or its amounts being below the test detection threshold).

Metagenomic next-generation sequencing (mNGS) is a promising approach for diagnosing infectious diseases since it can overcome some of the challenges faced by conventional diagnostic techniques such as PCR. For instance, mNGS has the potential to capture genomic information of any pathogen present in a biological sample, thereby saving the need for a broad panel of pathogen-specific tests. Due to its unbiased nature, mNGS can also identify novel (to a specific region or population), or highly divergent pathogens from those previously known, including new recombinant forms, or forms presenting atypical neurological manifestations [6]. In addition, mNGS provides pathogen genomic data, a resource increasingly leveraged in Public Health.

In this study, we used mNGS to retrospectively analyse leftover CSF samples from patients with aseptic meningitis in southern Spain (Andalusia region) for whom no cause had been found after routine clinical testing.

## Methods

### Sample selection and laboratory methods

Clinical CSF specimens used in this study were collected for routine clinical care from patients with suspected viral CNS infection in the Department of Neurology in the University Hospital Reina Sofia (Cordoba, Spain) between 2015 and 2019.

The Department of Neurology is responsible for the treatment of patients aged 14 years and older. It is the reference unit for patients with suspected CNS infections in the Córdoba province, where the number of inhabitants during the study period ranged from 795,611 in 2015 to 782,979 in 2019 [7]. CSF samples were obtained at patient admission according to standard procedures. For these CSF samples, protein and glucose levels were quantified, and white and red blood cells (WBC and RBC, respectively) were counted. Testing for a range of pathogenic agents according to the clinical suspicion of the attending physician was performed in the Unit of Clinical Microbiology. Included tested pathogens were enteroviruses (EVs), herpesviruses (cytomegalovirus (CMV), Epstein–Barr virus (EBV), herpes simplex viruses type 1 and type 2, human herpes virus type 6 and varicella zoster virus (VZV)), as well as bacterial pathogens (*Haemophilus influenzae type b*, *Listeria, Mycobacterium tuberculosis, Neisseria meningitidis* and *Streptococcus pneumoniae*). For some patients, serum samples were also obtained. In this case, these were screened for *Borrelia, Brucella, Coxiella, Cryptococcus*, *Rickettsia,* hepatitis viruses, HIV*, Mycoplasma pneumoniae,* parvovirus and *Treponema pallidum*. For patients with clinical suspicion for mumps virus (MuV), Toscana virus (TOSV) or West Nile virus (WNV), CSF was also tested for these viruses in the Virus Reference Laboratory of Andalucia at the University Hospital Virgen de las Nieves (Granada, Spain).

Of note, in Spain as in the EU, infections by TOSV are not notifiable and there is no case definition for TOSV. Testing is considered in patients with enterovirus- and herpesvirus-negative aseptic meningitis recalling recent insect bites. Other epidemiological factors such as summer season or rural residence may also be considered.

Sex, age, location of residence (rural or urban), month of sample collection, reporting of insect bites, length of hospital stay and clinical parameters were recorded. Information on routine laboratory testing performed in all selected patients (in CSF and serum samples) is provided in Supplementary Table S1. The laboratory methods used are summarised in Supplementary Table S2.

**Figure 1:**
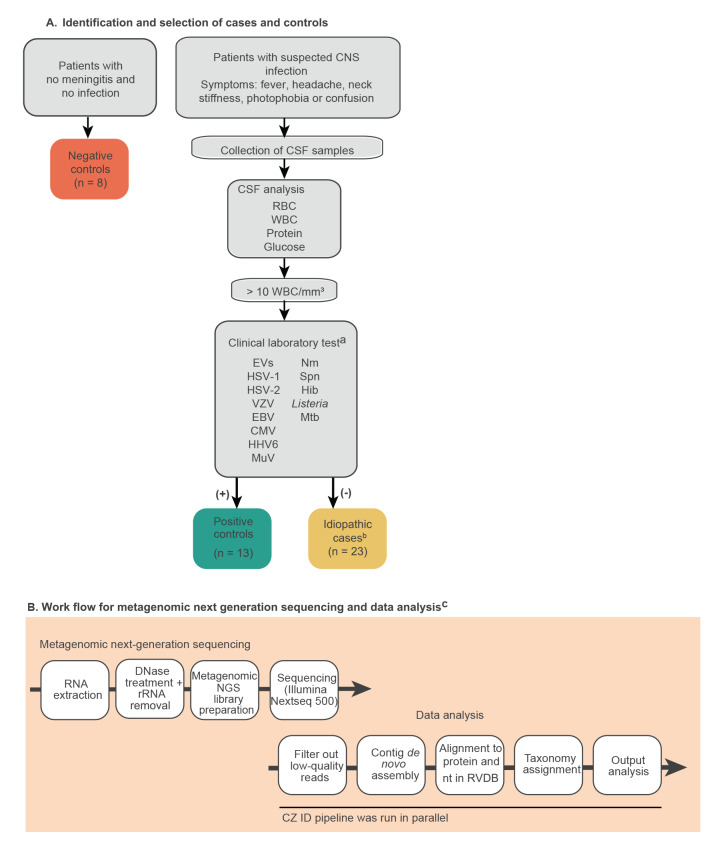
(A) Criteria to classify patients as negative or positive controls, or as idiopathic cases, and (B) workflow for metagenomic sequencing and analysis, Andalusia, Spain, 2015−2019 (n = 44 samples analysed)

CNS: central nervous system; CSF: cerebrospinal fluid; CZ-ID: Chan Zuckerberg ID; NGS: next generation sequencing; RBC: red blood cells; rRNA: ribosomal ribonucleic acid; RVDB: reference virus database; RT-qPCR: reverse transcription-quantitative PCR; TOSV: Toscana virus; WBC: white blood cells.

^a^ CMV: cytomegalovirus; EBV: Epstein–Barr virus; EVs: enteroviruses; HH6: human herpesvirus type 6; Hib: *Haemophilus influenzae* type b; HSV-1:  herpes simplex virus 1; HSV-2: herpes simplex virus 2; Mtb: *Mycobacterium tuberculosis*; MuV: mumps virus; Nm: *Neisseria meningitidis*; Spn: *Streptococcus pneumoniae*; VZV: varicella zoster virus.

^b^ Among the idiopathic cases, one sample (LCR_1152) had been prior tested for TOSV by RT-qPCR, but this had yielded a negative result. This sample was then shown by metagenomic sequencing to be positive for TOSV.

^c^ Data were analysed using custom scripts and tools on the CZ ID platform (https://czid;org/).

Residual CSFs were stored at − 45 ºC and those with sufficient volume (1 mL) were selected. Samples included in this study were:  (i) positive controls, which were randomly chosen CSF samples from patients with meningitis for whom the causative pathogen was previously identified by quantitative (q)PCR test; (ii) negative controls, which were randomly chosen CSF samples from patients with no meningitis and no infection but in whom another diagnosis was made (epilepsy or cognitive impairment); and (iii) idiopathic samples which were randomly chosen CSF samples from patients diagnosed with aseptic meningitis with unknown aetiological agent after conventional routine laboratory testing in the hospital (Figure 1). Meningitis-suspected cases were identified as described previously [8].

### Metagenomic next generation sequencing

The sample processing for mNGS and subsequent data analysis are described in the Supplementary Material. Briefly, the samples for mNGS underwent a ribosomal RNA depletion step prior to randomly primed reverse transcription and library preparation (Figure 1). Samples were sequenced on an Illumina NextSeq500, with a median depth of 11.7 million reads/sample (interquartile range (IQR): 9.9–16.5 million). After filtering out human and low-quality reads, the resulting FASTQ files were processed using an in-house pipeline (Supplementary Material), as well as on the CZ ID platform (https://czid.org/), a cloud-based open-source tool for pathogen detection from metagenomic data [9].

### Phylogenetic analysis

To build the TOSV dataset for phylogenetic reconstruction, the set of sequences generated during this study were combined with a set of partial and full-length sequences available in Virus Pathogen Resource (ViPR) [10] and GenBank [11] up to June 2022; the latter sequence set included representatives of the different TOSV genotypes. Partial sequences of less than 190 bp in length were excluded. We put together a total of 112, 88, and 80 sequences for the small (S), medium (M), and large (L) segments of the TOSV genome, respectively. The resulting dataset was aligned using Multiple Alignment using Fast Fourier Transform (MAFFT) v7.467 [12] for each segment separately and alignments were visually inspected in Geneious Prime 2020.2. We inferred maximum-likelihood (ML) phylogenies using IQ-TREE v2.0.6 [13]. Tree reconstruction was performed using the default settings and the best-fitting model provided by ModelFinder [14], followed by Ultrafast Bootstrap Approximation with 1,000 replicates [15] as implemented in IQ-TREE 2.

We similarly retrieved from ViPR all full-length MuV genome sequences available from 2008 up to October 2021 [10] and used these as well as the different World Health Organization (WHO) MuV genotype reference strain sequences to analyse the complete genome generated by mGNS in this study. This dataset containing a total of 211 sequences was employed to infer the ML phylogeny.

### Recombination analysis

Recombination analyses were carried out on concatenated genomes using the three segments of TOSV. A total of 29 genomes, including the seven generated in this study, were included in the analysis as described in Supplementary Table S3. We studied recombination using a combination of six methods (RDP, GENECONV, MaxChi, Bootscan, SisScan and 3SEQ) implemented in Recombination Detection Program (RDP)4 [16], and we considered recombination signals detected by more than three methods for breakpoint identification. Similarity plot and bootscanning analysis were performed using SimPlot v3.5.1 [17], with a 400-nt window moving in 40-nt steps and using a Kimura 2-parameter method with a transitions-transversions ratio of two with 1,000 resampling.

## Results

### Patient characteristics

The sample cohort consisted of positive controls (enteroviral meningitis, n = 12 and mumps meningitis, n = 1); negative controls (n = 8); and idiopathic meningitis samples (n = 23). All patients originated from Cordoba province and the majority (26/44) lived in large urban centres, while the remaining patients lived in villages (populations between 900 and 9,000 inhabitants). The age of the patients (n = 44, 26 male, 18 female) ranged from 14 to 95 years (mean: 41 years). Among the 23 idiopathic cases (17/23 male), 16 resided in urban areas. The age of the 23 cases ranged from 14 to 83 years (mean: 42 years), nine reported no insect bites when asked, and two explicitly mentioned insect bites, and for the 12 remainder this information was not available on the clinical record. 

**Figure 2:**
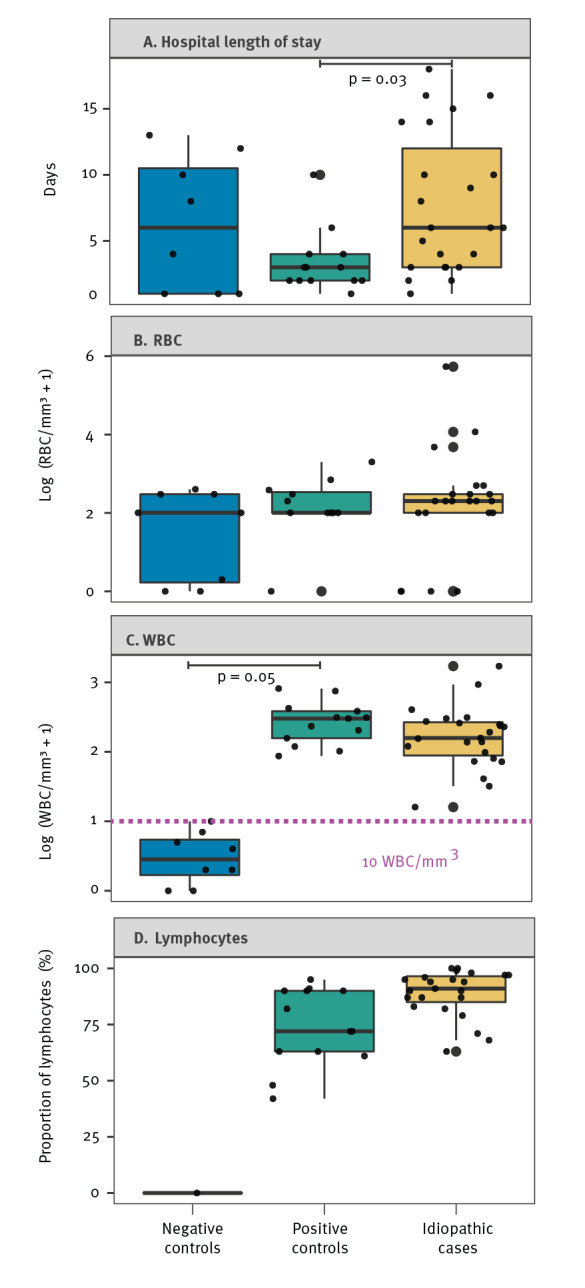
Respective comparison of (A) hospital length of stay and (B–C) blood cell abundance in cerebrospinal fluid samples from negative controls, positive controls and idiopathic aseptic meningitis cases, Andalusia, Spain, 2015−2019 (n = 44 samples analysed)

ANOVA: analysis of variance; CSF: cerebrospinal fluid; RBC: red blood cells; RTqPCR: reverse-transcription quantitative PCR; TOSV: Toscana virus; WBC: white blood cells.

The WBC was higher in idiopathic (median: 157.0 cells/mm^3^; IQR: 88.0–265.5 cells/mm^3^; norm adults: < 5 cells/mm^3^ of which lymphocytes 60–70%) and positive control (median: 300.0 cells/mm^3^; IQR: 156.0–383.0 cells/mm^3^) cases than in negative control cases (median: 2.0 cells/mm^3^; IQR: 0.8–5 cells/mm^3^). However, this difference was not statistically different (Figure 2). Lymphocyte predominance was observed in idiopathic (median: 91.0%; IQR: 85.0–96.5%) and positive control (median: 73.8%; IQR: 63.0–90.0%) CSF samples. Epidemiological and clinical characteristics recorded with the CSF samples and routine laboratory testing performed are provided in Supplementary Tables S1 and S2.

Comparison of characteristics of clinical samples among the cases (yellow) and two types of controls (positive (green) and negative (blue)) are shown in the Figure. Significantly different samples tested with one-way ANOVA with Tukey’s post-hoc test are indicated with the p value, p. The lymphocyte count was only available for one of the eight negative controls; no statistical test was performed. Norm RBC in CSF adults: 0 cells/mm^3^; norm WBC in CSF adults: < 5 cells/mm^3^ of which lymphocytes 60–70%.

### Metagenomic sequencing

In positive-control samples, we identified complete EV (n = 12) [8] and MuV (n = 1) genomes by mNGS, in accordance with prior diagnosis. In the negative control samples, neither approach revealed potential pathogens in the sequencing data. Surprisingly, among the 23 idiopathic cases with unknown aetiology, we detected TOSV genomic material in eight cases. TOSV had only been prior suspected for one (sample LCR_1152) of these TOSV-positive cases, however, the TOSV reverse-transcription (RT)-qPCR ordered during hospitalisation for this sample was negative. To rule out possible cross-contamination with another TOSV-positive sample, this sample was processed independently twice from the RNA extraction step. We reconstructed partial to almost complete segments (between 40–97% of the genome covered) for seven TOSV-positive samples, and only a partial fragment of the S and L segments for one sample as shown in Supplementary Table S4 and Figure S1. No pathogen could be identified from the rest of the idiopathic samples (n = 15).

### Characteristics of the mumps virus positive sample and phylogenetic analysis

**Figure 3:**
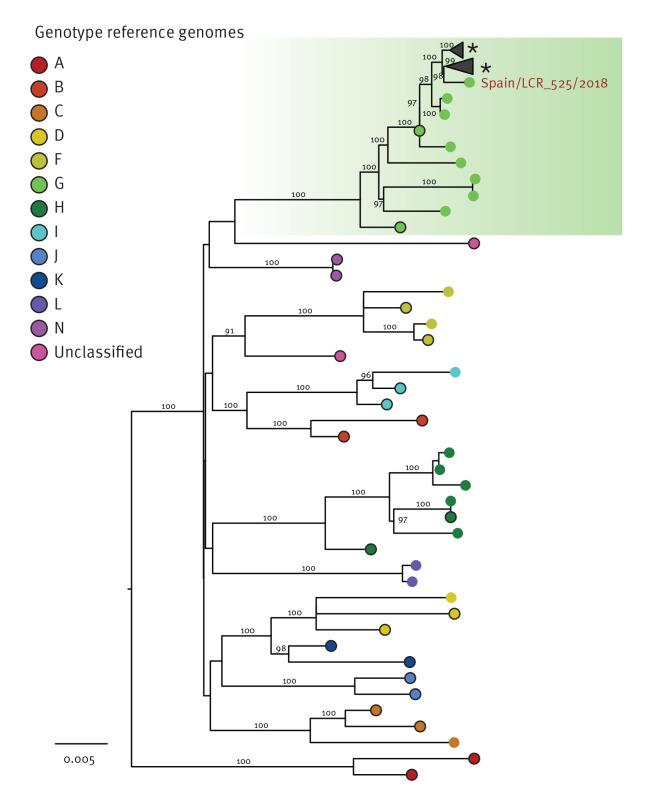
Maximum likelihood phylogenetic analysis of a full-length mumps virus genome sequence with other mumps virus sequences including those of the WHO reference strains for the different genotypes, Andalusia, Spain, 2018 (n = 211 sequences)

MuV: mumps virus; WHO: World Health Organization.

The positive control CSF sample found with MuV (LCR_525) was used to verify the capacity of mNGS, in our setting, to obtain exploitable genomic data. The sample had been obtained from a woman admitted to the hospital with parotitis, severe headache, and photophobia. The CSF specimen contained 747 WBC/mm^3^ and had tested positive for MuV by an in-house RT-qPCR [18]. mNGS corroborated this diagnosis and allowed to reconstruct a near-complete MuV genome sequence. This genome sequence was subsequently phylogenetically analysed using a dataset of 210 publicly available full-length MuV genome sequences, including those of the WHO reference genomes for the different MuV genotypes (A–N). The MuV/Spain/LCR_525/2018 genome derived from the LCR_525 sample clustered within genotype G, together with sequences collected from the United States (US) during 2015–2017 and Canada in 2017. The two sequences immediately basal to MuV/Spain/LCR_525/2018 are genomes collected in the US in 2011 (GenBank accession number: KY969482) and the Netherlands in 2010 (GenBank accession number: MW261742) (Figure 3). This is also illustrated in Supplementary Figure S2.

The tree leaves have circles filled with colour according to the MuV genotype. A black circle around the coloured tip denotes a WHO reference strain. The light green box highlights the MuV genotype G, the genotype to which the genome generated in this study (MuV/Spain/LCR_525/2018) belongs. The collapsed clusters with the asterisks correspond to sequences from the United States (2015–2017) and Canada (2017). Ultrafast bootstrap values above 80% are indicated above the branches leading to corresponding nodes. The scale bar indicates nucleotide substitutions per site. The tree is midpoint-rooted for visualisation. A complete expanded tree is shown in Supplementary Figure S2.

### Toscana virus amplicon-based sequencing

As mNGS only allowed to reconstruct partial genome sequences from some idiopathic cases, we built a PCR panel to improve coverage, as follows. Using the information obtained from mNGS, we designed a short PCR amplicon panel based on Quick et al. [19] targeting the known diversity of TOSV genotype B using Primal Scheme (https://primalscheme.com/ ). To test and optimise the panel, we used TOSV genomic RNA obtained from the European Virus Archive–Global (https://www.european-virus-archive.com/, Supplementary material). The optimised PCR panel can be found at https://github.com/Simon-LoriereLab/TOSV and in Supplementary Table S5. Using the targeted approach, we were able to generate almost complete TOSV genome sequences for seven of eight samples as shown in Supplementary Table S6 and Figure S3.

### Characteristics of patients testing positive for Toscana virus

All TOSV-infected patients were male. The age of patients ranged from 15 to 78 years (median: 39 years). The length of the hospital stay ranged from 2 to 16 days (median: 7 days). All these cases were detected between July and November in different years, compatible with the seasonal activity of sandflies in Mediterranean countries [20]. These patients were admitted to the hospital with severe headache and fever (≥ 38 °C), and their CSF contained high levels of WBC (median: 194.5 cells/mm^3^; IQR: 127.8–252.8 cells/mm^3^). Some of them also experienced vomiting, sensitivity to light and neck stiffness (Supplementary Table S1). All TOSV-positive patients reported no insect bites to the physician, except for one patient (sample 654) who reported insect bites during a trip to Portugal. Finally, the majority (5/8) lived in Cordoba city, a large urban centre of over 300,000 inhabitants.

### Phylogenetic analysis of Toscana virus

**Figure 4:**
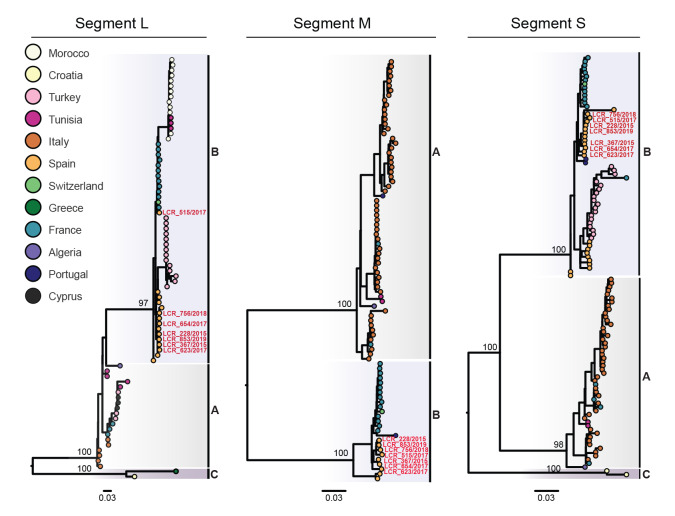
Maximum likelihood phylogenetic analysis of segment L, segment M and segment S of Toscana virus sequences generated in this study, Andalusia, Spain, 2015–2019 (n = 7 novel samples analysed)

In combination with a set of publicly available partial and complete TOSV sequences corresponding to the three TOSV genotypes (A, B and C), we generated ML phylogenies for each genome segment separately. The estimated phylogenies placed the novel sequences within genotype B with high support for the three segments (node support of 100, 100 and 97% for segments S, M and L, respectively) (Figure 4). Genotype B comprises sequences collected from France (2004–2015), Morocco (2008-2011, partial genomes), Portugal (1983), Spain (1998–2004), Switzerland (2018), Tunisia (2012, partial genomes) and Turkey (2013–2018, partial genomes), as can be viewed in Supplementary Figures S4–6. 

The maximum likelihood phylogenetic trees were inferred using IQ-TREE 2. Genotype A sequences are highlighted in grey, B in blue, and C in purple. Novel genomes reported here are noted in red. The circles at the branch tips are coloured according to country. Branch support values for the main lineages are ultrafast bootstrap percentages, and the scale bars represent the number of nucleotide substitutions per site. Trees with full taxa names are provided in Supplementary Figures S4–6.

**Figure 5:**
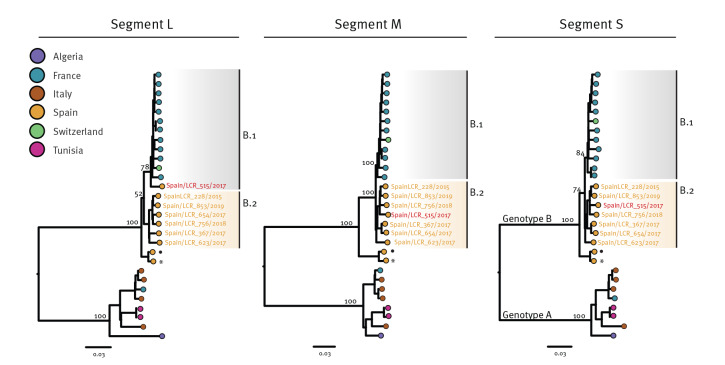
Phylogenetic analysis of complete Toscana virus genome sequences, including those generated in this study, highlighting the incongruent position of the TOSV/Spain/LCR_515/2017 strain sequence, Andalusia, Spain, 2015–2019 TOSV (n = 29 sequences)

Phylogenies inferred using only complete or almost complete genomes showed similar topologies, where genotype B may be consistently subdivided into two lineages, B.1 and B.2 (Figure 5 and Supplementary Figures S4, S5 and S6). While lineage B.1 contained sequences collected from France, Morocco, Switzerland or Tunisia, lineage B.2 included sequences from Spain and Turkey suggesting that the circulation of the distinct viral lineages may be segregated between these two sets of countries, with some countries appearing to have different lineages despite their geographic proximity. In a phylogenetic tree based on complete genome information (Figure 5), two sequences collected from Spain in 1993–1999 (strain ESH_62100) and 2003–2004 (strain EsPhGR40) appeared basal to the two groups forming B.1 and B.2 lineages [21].

TOSV: Toscana virus.

All available complete TOSV genomes (n = 22) were combined with the genomes generated in this study (n = 7) to infer maximum likelihood trees using IQ-TREE 2. Proposed lineages B.1 and B.2 within genotype B are highlighted. Strains ESH_62100 and EsPhGR40, basal to B.1 and B.2 in this analysis are noted with an asterisk and a dot symbol, respectively. The numbers at the nodes indicate bootstrap support values in per cent. The scale bar represents nucleotide substitutions per site. Taxa names and metadata of each genome are provided in Supplementary Table S3. Phylogenies can be visualised interactively at https://github.com/Simon-LoriereLab/TOSV.

Interestingly, the phylogenetic placement of sample TOSV/Spain/LCR_515/2017 from one of the study cases did not seem consistent between segment L and the other segments analyses, suggesting intra-genotype reassortment. Indeed, the L segment of sample TOSV/Spain/LCR_515/2017 appears within the lineage B.1 cluster, whereas both segments M and S figure within the lineage B.2 cluster. To further explore this incongruence, we performed a recombination analysis using concatenated genomes of the TOSV sequences and a combination of models implemented in RDP4. The concatenated TOSV/Spain/LCR_515/2017 genome presented strong signal of recombination in five different methods including 3SEQ. Similarly, similarity plot and bootscanning analysis showed higher similarity of TOSV/Spain/LCR_515/2017 to B.2 lineage across the S and M segment region and higher similarity to B.1 sequences across the L segment, further supporting an intra-genotype reassortment event; this is illustrated in Supplementary Figure S7.

## Discussion

In this study, we used mNGS on CSF samples from aseptic meningitis cases in southern Spain with unknown aetiology. To validate our methodology, we included positive and negative control samples. Our work highlights the value of mNGS as a potent diagnostic tool as we confirmed viruses that had been previously identified by conventional PCR in all positive controls (EV, n = 12 and MuV, n = 1 genomes, two RNA viruses well-known to cause meningitis). In addition, our approach allowed to reconstruct complete genomes from these positive controls, thus providing useful molecular epidemiology data such as the first whole-genome sequence of MuV in Spain or the identification of a novel Echovirus 13 recombinant form [8]. In this study we detected TOSV genetic material in eight of 23 idiopathic aseptic meningitis cases, a rate of identification of an aetiology similar to the one reported in the study from Saha et al. (40%) in Bangladesh using a similar dataset (idiopathic meningitis; n = 25) and a similar RNA mNGS approach on CSF [22].

Interestingly, although based on smaller numbers of patients, the frequency of TOSV detection appeared notably higher than observed in previous retrospective CSF studies in the region of Madrid and southern Spain between 1998 and 2013, where 58 of 1,393 idiopathic aseptic meningitis cases (4.2%) were identified as TOSV cases by PCR [23]. Such results could be attributed to the enhanced viral detection capability of mNGS over conventional testing alone as reported in previous studies [24,25], or higher incidence of TOSV in the region during the study period.

As the mNGS failed to generate full-length TOSV genomes for a fraction of samples (4/8 samples, Supplementary Table S4), we developed and optimised a highly multiplexed PCR amplicon panel, using an approach known to help with challenging samples (low viral load or degraded RNA) [19]. Using this amplicon panel, we obtained almost complete sequences from all but one identified sample (Spain/LCR_1152/2019). This sample was the only one that had been prior tested for TOSV by RT-qPCR, with a negative result. Although none of the CSF samples contained high amounts of TOSV RNAs, to address the risk of cross-contamination, this sample was processed a second time by a different experimenter in different facilities starting from the RNA extraction and the result did not change. A possible explanation for the discrepancy between mNGS and RT-qPCR could be that TOSV RNAs were too degraded for RT-qPCR detection. Indeed, the RT-qPCR target region – among others – was not recovered by the sequencing of this sample. This result highlights the strength of mNGS to detect pathogens in clinical specimens even with degraded samples.

TOSV is a phlebovirus transmitted by sandflies of the genus *Phlebotomus* [26], and it has been detected in many countries surrounding the Mediterranean Sea. Although seroprevalence studies suggest that TOSV causes asymptomatic infections in most cases, some individuals experience severe disease with fever, intense headache, vomiting, or neurological diseases such as meningitis or encephalitis. Indeed, a recent study showed that TOSV is the most common cause of summer viral meningitis in central Italy, with meningitis cases by TOSV outnumbering those by EV [27]. In Spain, sporadic meningitis cases and series of TOSV infections have been documented in the Andalusia region, the Mediterranean coast and Madrid [23,28]. In Andalusia, where Cordoba study province is located, 107 TOSV positive cases have been diagnosed from 1988 to 2020 and the region is considered to be endemic for TOSV [29]. Despite its recognised clinical importance, TOSV remains a neglected virus [26]. In Spain, cases are often identified retrospectively in research studies indicating a lack of clinical suspicion for TOSV in patients with meningitis [23]. Only one patient in the current study cohort (LCR_1152) had been tested for TOSV by clinicians. This patient did not report recent insect bites, but had an outdoor occupation associated with a higher risk for TOSV infection [29]. The lack of suspicion for TOSV may lie in the rare report of insect bites (only one TOSV positive patient mentioned insect bites to the doctor when asked). The difficulty in remembering insect bites could be explained by the long incubation period for TOSV neuroinvasive infection, estimated at 12 days (95% confidence interval (CI): 10.2–14.4) [30]. It is also noteworthy that the majority (5/8) of TOSV positive cases identified here indicated living in large urban centres which differed from previous studies reporting most TOSV cases in rural areas in Spain [23]. This difference may reflect an evolution of the geographic distribution of sandflies, impacting on the epidemiology of associated human and animal pathogens such as TOSV [31]. Additional studies, including detailed patient information (e.g. employment, outdoor activities, out-of-town secondary residences) would help to evaluate the factors associated with higher risk of TOSV infection. Finally, some TOSV positive cases (3/8) corresponded to infections that occurred during autumn months (September–November). Although detection of TOSV in Spain outside of summertime has been sporadically reported [23], it is important to raise awareness among clinicians on this potential expansion of temporality. Overall, our study calls for considering the inclusion of TOSV in the differential diagnosis of patients presenting with enterovirus- and herpesvirus-negative aseptic meningitis regardless of history of insect bites mentioned by the patient, summer season or rural residence. 

Another reason contributing to the neglected nature of this virus is the lack of laboratory testing for TOSV in some Mediterranean countries. In Andalusia, most hospital laboratories do not offer testing, and TOSV-suspected samples are sent to the Regional Reference Virology Laboratory in Granada, possibly delaying diagnosis. Including TOSV in the routine diagnosis of aseptic meningitis cases at the hospitals in southern Spain could result in shortened length of hospital stay (median 7 days in this study for TOSV-positive patients), reduced associated costs [3], and improved patient care. The recurrent detection of TOSV across the Mediterranean region [32] and the detection of TOSV and its vector in European countries outside this region, such as Germany (2006–2016, n = 4) [33], calls for increased TOSV awareness and vigilance in Europe. Importantly, the timely identification of a vector-borne infection is crucial for public health risk management strategies such as local vector control programmes.

Analysis of the complete TOSV genomes provides evidence of intra-genotype reassortment involving the L segment. The mechanism of reassortment between different viruses within the *Phlebovirus* genus has been widely described [34], consisting in all cases of the replacement of the M segment [35,36]. This TOSV reassortment has multiple implications. Indeed, while the genomic data previously available for the B.1 and B.2 lineages suggest a strong geographical clustering within genotype B, this genomic exchange indicates that both lineages must co-circulate or have co-circulated in recent years, in Spain or the South of Europe. This also suggests that TOSV prevalence – and genetic diversity – in its reservoir or vector populations is underestimated. We hope that the targeted approach developed here, together with the ongoing increase in pathogen genomic sequencing efforts, will help enrich the databases (including completing partial genomes), allowing high-resolution phylodynamic analysis to better understand the epidemiology and evolution of this virus.

Our work has several limitations. The storage conditions of the samples used in this study were not optimal:  the samples were collected between 2015 and 2019, stored at − 45 °C until processed in 2019, and subjected to multiple freeze-thaw cycles. This fact could have affected the quality of the RNA, contributing to the proportion of cases that remained unsolved. In addition, our metagenomic approach focuses on RNA so, in terms of using this method for DNA pathogens, while the mRNA of such pathogens can be potentially detected [37], if these replicate at a low level, they might go undetected.

In the Andalusia Region, aseptic meningitis is a mandatorily and urgently notifiable disease. From 2016 to 2017, a total of 567 cases of viral meningitis were reported in Andalusia of which 275 (48.5%) remained with an undiagnosed aetiology [29]. In conclusion, to reduce these numbers, our work calls for (i) testing patients presenting with enterovirus- and herpesvirus-negative aseptic meningitis in Andalusia for TOSV regardless of their recalling of insect bites, summer season or rural living and (ii) including TOSV as a target in neurological panels in the routine laboratory-testing at hospitals in southern Spain. Finally, this work demonstrates the utility of mNGS to identify viral pathogens directly in CSF and provides a methodological improvement to facilitate TOSV full-genome sequencing [32]. The detection of a novel, disease-associated, reassortant TOSV emphasises the importance of full-genome surveillance to monitor the spread and evolution of phleboviruses.

## Supplementary Data

8081000 Supplementary Material
