# Supplementary material for: Untargeted metagenomic sequencing identifies Toscana virus in patients with idiopathic meningitis, southern Spain, 2015 to 2019
Source: Euro Surveill. 2023 Nov 9;28(45):2200913. doi: 10.2807/1560-7917.ES.2023.28.45.2200913 (PMC10636744; doi:10.2807/1560-7917.ES.2023.28.45.2200913)
Supplement: Supplementary Material [file 2200913_SupplementaryMaterial.pdf]

Supplementary material

This supplementary material is hosted by Eurosurveillance as supporting information alongside the article "Untargeted metagenomic sequencing identifies Toscana virus in patients with idiopathic meningitis, Southern Spain, 2015 to 2019", on behalf of the authors, who remain responsible for the accuracy and appropriateness of the content. The same standards for ethics, copyright, attributions and permissions as for the article apply. Supplements are not edited by Eurosurveillance and the journal is not responsible for the maintenance of any links or email addresses provided therein.

Table of contents

Methods

References

Figures and tables legends

Methods

RNA extraction

RNA was extracted from 140 ul of CSF using the QIAamp Viral RNA Mini Kit (Qiagen) following the manufacturer's recommendations and including the RNA carrier. This was followed by Turbo DNase treatment (Ambion) and purification with Agencourt RNAClean XP beads (Beckman Coulter).

Metagenomic next-generation sequencing

We used a previously described protocol for untargeted metagenomic sequencing of clinical samples [1]. Briefly, prior to library construction, poly-A carrier RNA and host ribosomal RNA were depleted using oligo (dT) and custom probes, respectively, to form RNase H target DNA-RNA hybrids. The RNA resulting from selective depletion was used for random-primed cDNA synthesis using the SuperScript IV (Invitrogen). Second-strand cDNA was generated using a cocktail of enzymes, including *Escherichia coli* DNA ligase, RNAse H, and DNA polymerase (New England Biolabs), and then purified using Agencourt AMPure XP beads (Beckman Coulter). We prepared libraries from the dsDNA using the Nextera XT kit (Illumina).

Metagenomic next-generation sequencing data analysis

Initial removal of human reads was performed with STAR 2.7.9 [2] and a second round of human reads filtering out was carried out with Bowtie2 [3]. The resulting fastq files were analyzed with CZID, a cloud-based metagenomic platform designed for the detection of microbes from metagenomic data [4]. Scripts and user instructions are available at https://github.com/chanzuckerberg/czid-dag. Briefly, adapter, low-quality reads and human host sequences are filtered out, and the remaining reads are aligned to the NCBI nucleotide (nt) and non-redundant protein (nr) and grouped in taxonomic bins. In parallel, reads are *de novo* assembled SPAdes v3.12 [5], and the contigs obtained were used to map back the binned reads from the previous step. Finally, each contig is aligned to a set of possible accession numbers represented by the BLAST database generated in the previous step. Potential pathogens were distinguished from commensal flora and contaminants from reagents or the environment by establishing a Z-score metric based on a background model derived from control samples. These samples included eight negative control samples (CSF from patients with no infection and no meningitis) and two "water-only" control samples. All taxa with Z-score less than 1, contig shorter than 35 base pairs, and number of reads per million less than 10 were removed from analysis.

In parallel, we also used an in-house pipeline to analyze the sequencing data. After quality trimming, raw reads were assembled in parallel with metaSPAdes v3.14 and megahit v1.2.9. The resulting contigs were used as queries for blastx using DIAMOND v2.0.14 against version 18.0 of the RVDB protein database [6] and the nr database. Taxonomy was assigned to each contig with DIAMOND and in-house R [7] scripts were used to analyze the resulting output. For the pathogens identified by DIAMOND blastx, chimeric contigs were constructed using the longest assembled contig and a reference genome. For TOSV, this was done for the three segments separately, and the reference genome used was a consensus sequence result of an alignment of TOSV genotype B sequences. For Mumps virus, we used the reference genome (GenBank ID: NC_002200). In all cases, nucleotide divergence between the contig and the reference genome was less than 2%. These chimeric contigs were used as scaffolds to map the reads, using clc-assembly-cell v5.1.0. The virus consensus sequence generation was performed with ivar v1.0 using a minimum of 5X read depth coverage. In case of lower read coverage, we added N. Samtools v1.10 [8] was used to sort the aligned BAM files and generate alignment statistics. We manually inspected all alignments and consensus sequences using Geneious Prime 2020.2 (https://www.geneious.com/).

Amplicon-based sequencing

To obtain complete TOSV genomes, we implemented a highly multiplexed short PCR amplicon approach [9]. The primer scheme was designed using PrimalScheme [10] to generate ~ 400 nucleotide long overlapping amplicons to cover the entire length of the three TOSV segments. The primers are divided into two separate primer pools (pool1 and pool2), generating non-overlapping amplicons pooled in the following protocol step to cover the entire genome. We followed the protocol generated by Quick, J. et al. [10] to generate the tiled virus amplicons. Briefly, two microliters of viral cDNA were used in the two multiplexed

PCR reactions using Q5 DNA High-fidelity Polymerase (New England Biolabs) to obtain ~ 400 nucleotide long amplicons in 35 cycles. Amplicons were purified using Agencourt AMPure XP beads (Beckman Coulter) and combined to 50ng. Libraries were constructed using the NexteraXT kit. Libraries quality and quantification were assessed using Qubit 4 (Thermo Fisher), Bioanalyzer (Agilent), and qPCR (NEBNext Library Quant Kit, Illumina) and sequenced using a paired-end strategy on an Illumina NextSeq500 platform (2x75 cycles).

Primer pool scheme optimization

We constructed three primer pool schemes that we tested and validated using a TOSV genomic standard for the genotype B (Toscana Standard#1, strain MRS2010 4319501) obtained from the European Viral Archive (https://www.european-virus-archive.com). We chose the primer pool that yielded the best coverage throughout the TOSV genome. The primer pool scheme and protocol can be found at https://github.com/Simon-LoriereLab/TOSV.

references

1. Matranga CB, Andersen KG, Winnicki S, Busby M, Gladden AD, Tewhey R, et al. Enhanced methods for unbiased deep sequencing of Lassa and Ebola RNA viruses from clinical and biological samples. Genome biology. 2014;15(11):1-12.
2. Dobin A, Davis CA, Schlesinger F, Drenkow J, Zaleski C, Jha S, et al. STAR: ultrafast universal RNA-seq aligner. Bioinformatics. 2013;29(1):15-21.
3. Langmead B, Salzberg SL. Fast gapped-read alignment with Bowtie 2. Nat Methods. 2012;9(4):357-9.
4. Kalantar KL, Carvalho T, de Bourcy CFA, Dimitrov B, Dingle G, Egger R, et al. IDseq-An open source cloud-based pipeline and analysis service for metagenomic pathogen detection and monitoring. Gigascience. 2020;9(10).
5. Nurk S, Meleshko D, Korobeynikov A, Pevzner PA. metaSPAdes: a new versatile metagenomic assembler. Genome research. 2017;27(5):824-34.
6. Bigot T, Temmam S, Pérot P, Eloit M. RVDB-prot, a reference viral protein database and its HMM profiles. F1000Research. 2020;8:530.
7. Ihaka R, Gentleman R. R: a language for data analysis and graphics. Journal of computational and graphical statistics. 1996;5(3):299-314.
8. Li H, Handsaker B, Wysoker A, Fennell T, Ruan J, Homer N, et al. The sequence alignment/map format and SAMtools. Bioinformatics. 2009;25(16):2078-9.
9. Grubaugh ND, Gangavarapu K, Quick J, Matteson NL, De Jesus JG, Main BJ, et al. An amplicon-based sequencing framework for accurately measuring intrahost virus diversity using PrimalSeq and iVar. Genome Biology. 2019;20(1).
10. Quick J, Grubaugh ND, Pullan ST, Claro IM, Smith AD, Gangavarapu K, et al. Multiplex PCR method for MinION and Illumina sequencing of Zika and other virus genomes directly from clinical samples. Nature Protocols. 2017;12(6):1261-76.

Figures and Tables legends

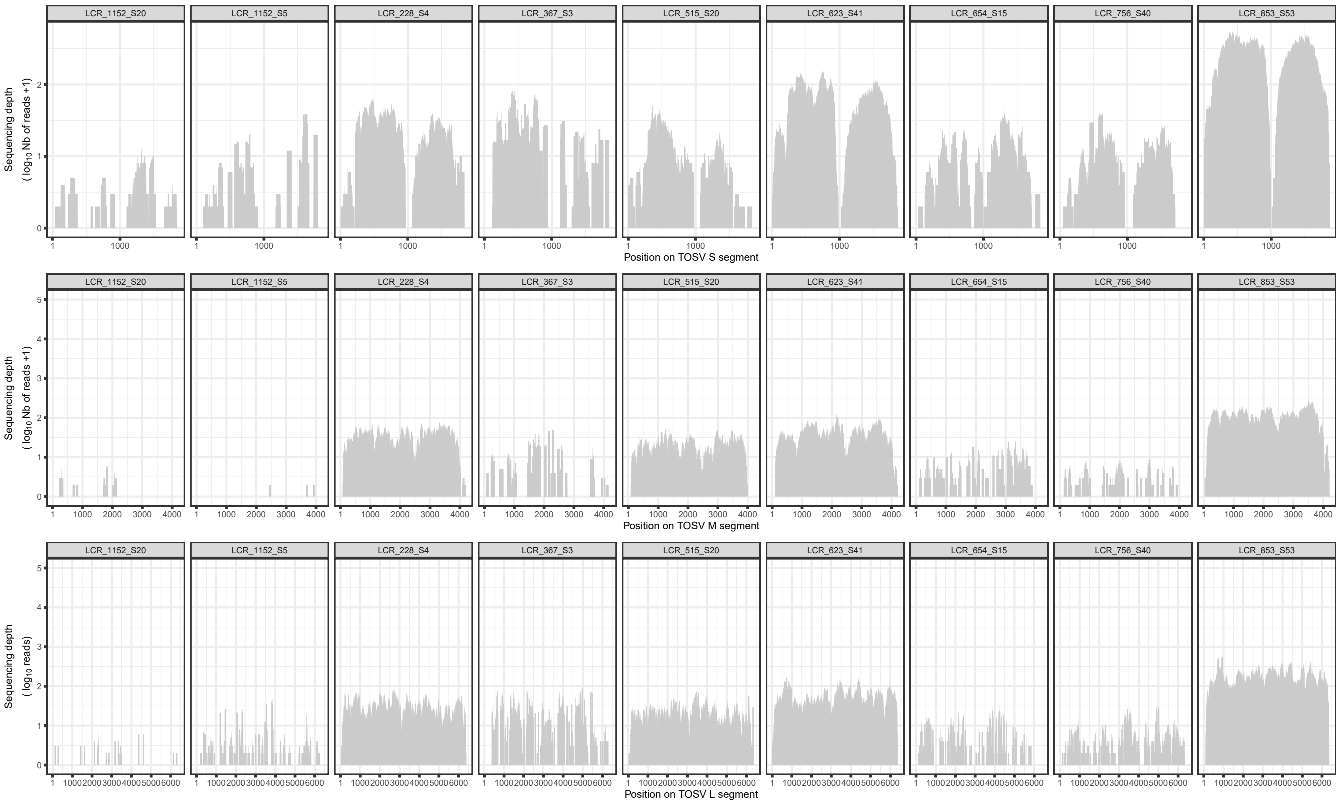
LCR_1152_S20
LCR_1152_S5
LCR_228_S4
LCR_367_S3
LCR_515_S20
LCR_623_S41
LCR_654_S15
LCR_756_S40
LCR_853_S53
Sequencing depth (log_{10} Nb of reads +1)
Position on TOSV S segment
Position on TOSV M segment
Sequencing depth (log_{10} reads)
Position on TOSV L segment

Figure S1: TOSV genome coverage for the three segments (L, M, and S) obtained using an untargeted metagenomic sequencing, n=8 samples analyzed, Andalusia, Spain, 2015-2019). The genome coverage is represented as the logarithm function of the total number of reads + 1 (to avoid conflicts with regions with zero coverage) for the eight different samples in which TOSV was detected. Sample LCR_1152 has been sequenced twice, and here we show the result of both runs: LCR_1152_S5 and LCR_1152_S20.

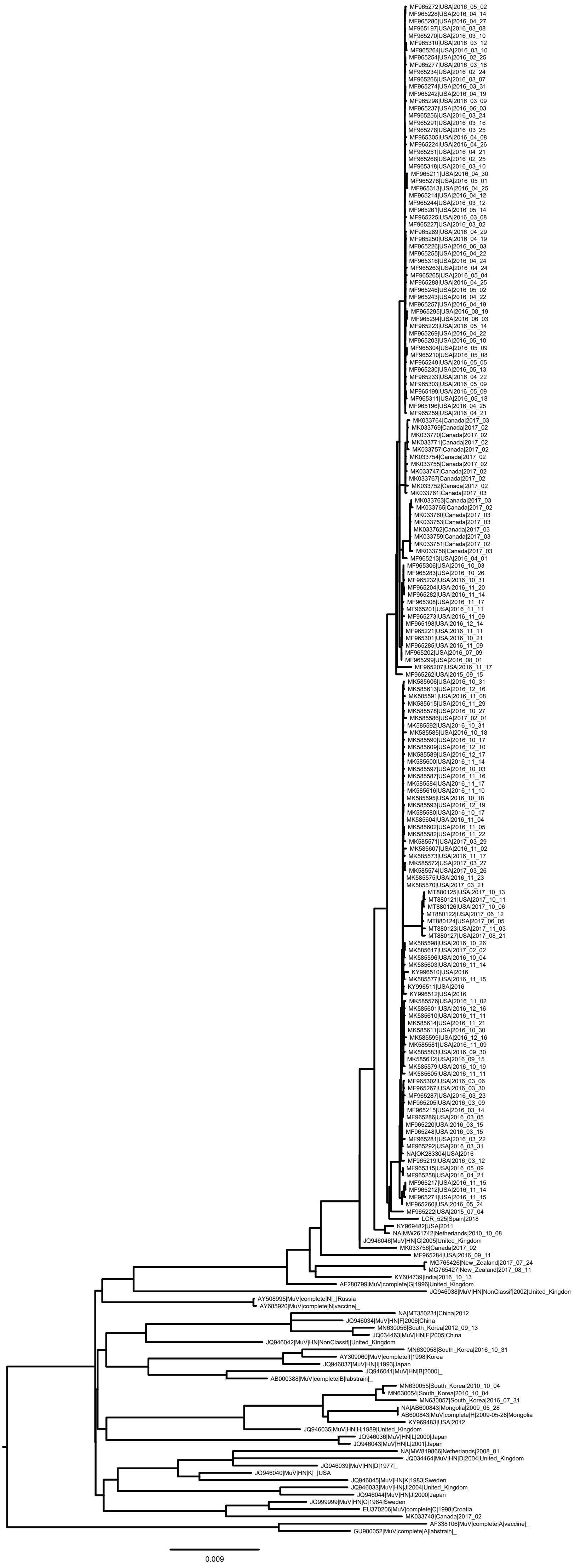
MF965272|USA|2016_05_02
MF965228|USA|2016_04_14
MF965280|USA|2016_04_27
MF965197|USA|2016_03_08
MF965270|USA|2016_03_10
MF965310|USA|2016_03_12
MF965264|USA|2016_03_10
MF965254|USA|2016_02_25
MF965277|USA|2016_03_18
MF965234|USA|2016_02_24
MF965266|USA|2016_03_07
MF965274|USA|2016_03_31
MF965242|USA|2016_04_19
MF965298|USA|2016_03_09
MF965237|USA|2016_06_03
MF965256|USA|2016_03_24
MF965291|USA|2016_03_16
MF965278|USA|2016_03_25
MF965305|USA|2016_04_08
MF965224|USA|2016_04_26
MF965251|USA|2016_04_21
MF965268|USA|2016_02_25
MF965318|USA|2016_03_10
MF965211|USA|2016_04_30
MF965276|USA|2016_05_01
MF965313|USA|2016_04_25
MF965214|USA|2016_04_12
MF965244|USA|2016_03_12
MF965261|USA|2016_05_14
MF965225|USA|2016_03_08
MF965227|USA|2016_03_02
MF965289|USA|2016_04_29
MF965250|USA|2016_04_19
MF965226|USA|2016_06_03
MF965255|USA|2016_04_22
MF965316|USA|2016_04_24
MF965263|USA|2016_04_24
MF965265|USA|2016_05_04
MF965288|USA|2016_04_25
MF965246|USA|2016_05_02
MF965243|USA|2016_04_22
MF965257|USA|2016_04_19
MF965295|USA|2016_08_19
MF965294|USA|2016_06_03
MF965223|USA|2016_05_14
MF965269|USA|2016_04_22
MF965203|USA|2016_05_10
MF965304|USA|2016_05_09
MF965210|USA|2016_05_08
MF965249|USA|2016_05_05
MF965230|USA|2016_05_13
MF965233|USA|2016_04_22
MF965303|USA|2016_05_09
MF965199|USA|2016_05_09
MF965311|USA|2016_05_18
MF965196|USA|2016_04_25
MF965259|USA|2016_04_21
MK033764|Canada|2017_03
MK033769|Canada|2017_02
MK033770|Canada|2017_02
MK033771|Canada|2017_02
MK033757|Canada|2017_02
MK033754|Canada|2017_02
MK033755|Canada|2017_02
MK033747|Canada|2017_02
MK033767|Canada|2017_02
MK033752|Canada|2017_02
MK033761|Canada|2017_03
MK033763|Canada|2017_03
MK033765|Canada|2017_02
MK033760|Canada|2017_03
MK033753|Canada|2017_03
MK033762|Canada|2017_03
MK033759|Canada|2017_03
MK033751|Canada|2017_02
MK033758|Canada|2017_03
MF965213|USA|2016_04_01
MF965306|USA|2016_10_03
MF965283|USA|2016_10_26
MF965232|USA|2016_10_31
MF965204|USA|2016_11_20
MF965282|USA|2016_11_14
MF965308|USA|2016_11_17
MF965201|USA|2016_11_11
MF965273|USA|2016_11_09
MF965198|USA|2016_12_14
MF965221|USA|2016_11_11
MF965301|USA|2016_10_21
MF965285|USA|2016_11_09
MF965202|USA|2016_07_09
MF965299|USA|2016_08_01
MF965207|USA|2016_11_17
MF965262|USA|2015_09_15
MK585606|USA|2016_10_31
MK585613|USA|2016_12_16
MK585591|USA|2016_11_08
MK585615|USA|2016_11_29
MK585578|USA|2016_10_27
MK585586|USA|2017_02_01
MK585592|USA|2016_10_31
MK585585|USA|2016_10_18
MK585590|USA|2016_10_17
MK585609|USA|2016_12_10
MK585589|USA|2016_12_17
MK585600|USA|2016_11_14
MK585597|USA|2016_10_03
MK585587|USA|2016_11_16
MK585584|USA|2016_11_17
MK585616|USA|2016_11_10
MK585595|USA|2016_10_18
MK585593|USA|2016_12_19
MK585580|USA|2016_10_17
MK585604|USA|2016_11_04
MK585602|USA|2016_11_05
MK585582|USA|2016_11_22
MK585571|USA|2017_03_29
MK585607|USA|2016_11_02
MK585573|USA|2016_11_17
MK585572|USA|2017_03_27
MK585574|USA|2017_03_26
MK585575|USA|2016_11_23
MK585570|USA|2017_03_21
MT880125|USA|2017_10_13
MT880121|USA|2017_10_11
MT880126|USA|2017_10_06
MT880122|USA|2017_06_12
MT880124|USA|2017_06_05
MT880123|USA|2017_11_03
MT880127|USA|2017_08_21
MK585598|USA|2016_10_26
MK585617|USA|2017_02_02
MK585596|USA|2016_10_04
MK585603|USA|2016_11_14
KY996510|USA|2016
MK585577|USA|2016_11_15
KY996511|USA|2016
KY996512|USA|2016
MK585576|USA|2016_11_02
MK585601|USA|2016_12_16
MK585610|USA|2016_11_11
MK585614|USA|2016_11_21
MK585611|USA|2016_10_30
MK585599|USA|2016_12_16
MK585581|USA|2016_11_09
MK585583|USA|2016_09_30
MK585612|USA|2016_09_15
MK585579|USA|2016_10_19
MK585605|USA|2016_11_11
MF965302|USA|2016_03_06
MF965267|USA|2016_03_30
MF965287|USA|2016_03_23
MF965205|USA|2016_03_09
MF965215|USA|2016_03_14
MF965286|USA|2016_03_05
MF965220|USA|2016_03_15
MF965248|USA|2016_03_15
MF965281|USA|2016_03_22
MF965292|USA|2016_03_31
NA|OK283304|USA|2016
MF965219|USA|2016_03_12
MF965315|USA|2016_05_09
MF965258|USA|2016_04_21
MF965217|USA|2016_11_15
MF965212|USA|2016_11_14
MF965271|USA|2016_11_15
MF965260|USA|2016_05_24
MF965222|USA|2015_07_04
LCR_525|Spain|2018
KY969482|USA|2011
NA|MW261742|Netherlands|2010_10_08
JQ946046|MuV|HN|G|2005|United_Kingdom
MK033756|Canada|2017_02
MF965284|USA|2016_09_11
MG765426|New_Zealand|2017_07_24
MG765427|New_Zealand|2017_08_11
KY604739|India|2016_10_13
AF280799|MuV|complete|G|1996|United_Kingdom
JQ946038|MuV|HN|NonClassif|2002|United_Kingdon
AY508995|MuV|complete|N|_|Russia
AY685920|MuV|complete|N|vaccine|_
NA|MT350231|China|2012
JQ946034|MuV|HN|F|2006|China
MN630056|South_Korea|2012_09_13
JQ034463|MuV|HN|F|2005|China
JQ946042|MuV|HN|NonClassif||United_Kingdom
MN630058|South_Korea|2016_10_31
AY309060|MuV|complete|I|1998|Korea
JQ946037|MuV|HN|I|1993|Japan
JQ946041|MuV|HN|B|2000|_
AB000388|MuV|complete|B|labstrain|_
MN630055|South_Korea|2010_10_04
MN630054|South_Korea|2010_10_04
MN630057|South_Korea|2016_07_31
NA|AB600843|Mongolia|2009_05_28
AB600843|MuV|complete|H|2009-05-28|Mongolia
KY969483|USA|2012
JQ946035|MuV|HN|H|1989|United_Kingdom
JQ946036|MuV|HN|L|2000|Japan
JQ946043|MuV|HN|L|2001|Japan
NA|MW819866|Netherlands|2008_01
JQ034464|MuV|HN|D|2004|United_Kingdom
JQ946039|MuV|HN|D|1977|_
JQ946040|MuV|HN|K|_|USA
JQ946045|MuV|HN|K|1983|Sweden
JQ946033|MuV|HN|J|2004|United_Kingdom
JQ946044|MuV|HN|J|2000|Japan
JQ999999|MuV|HN|C|1984|Sweden
EU370206|MuV|complete|C|1998|Croatia
MK033748|Canada|2017_02
AF338106|MuV|complete|A|vaccine|_
GU980052|MuV|complete|A|labstrain|_
0.009

Figure S2: Phylogenetic divergence tree of all labeled MuV sequences used. The maximum-likelihood tree includes MuV full-length genomes retrieved from GenBank in October 2021, the WHO reference strains for the different genotypes, and the MuV genome generated in this study (Spain/LCR_525/2018), Andalusia, Spain, 2018.

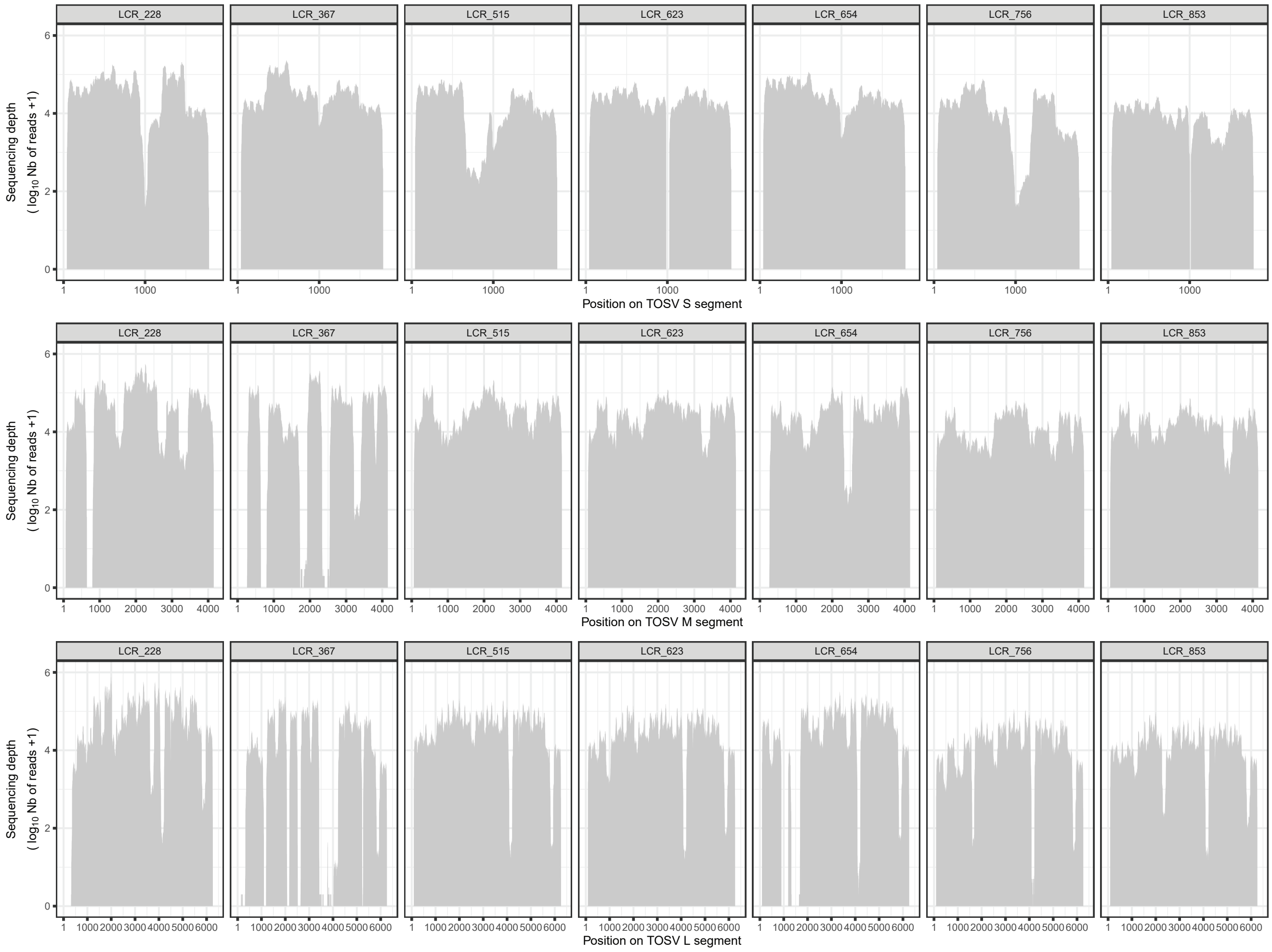
LCR_228
LCR_367
LCR_515
LCR_623
LCR_654
LCR_756
LCR_853
Sequencing depth
($\log_{10}$ Nb of reads +1)
Position on TOSV S segment
Position on TOSV M segment
Position on TOSV L segment

Figure S3: TOSV genome coverage for the three segments (L, M, and S) obtained applying an amplicon-based sequencing approach, n=8 samples analyzed, Andalusia, Spain 2015-2019.

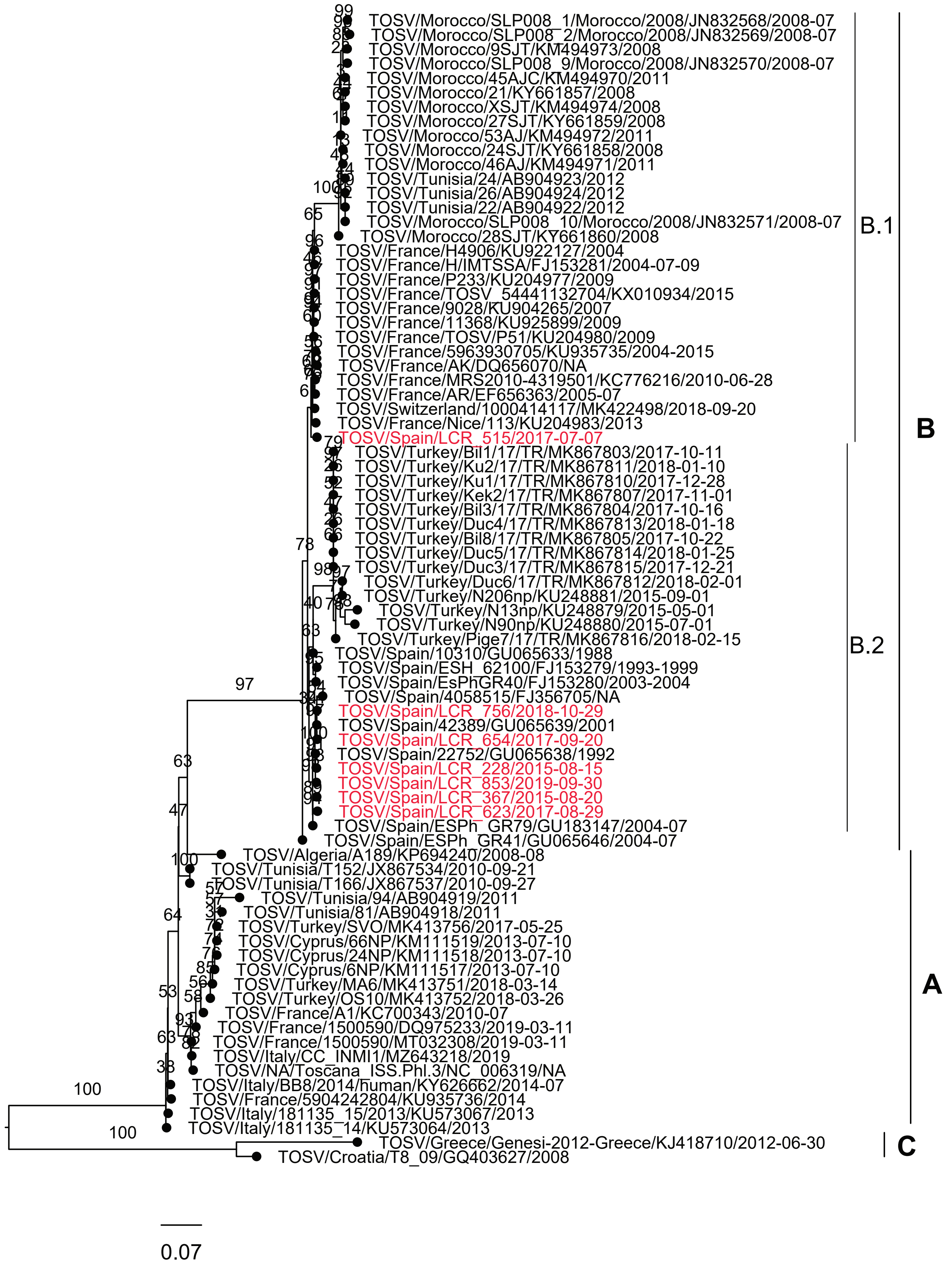

TOSV/Morocco/SLP008_1/Morocco/2008/JN832568/2008-07
TOSV/Morocco/SLP008_2/Morocco/2008/JN832569/2008-07
TOSV/Morocco/9SJT/KM494973/2008
TOSV/Morocco/SLP008_9/Morocco/2008/JN832570/2008-07
TOSV/Morocco/45AJC/KM494970/2011
TOSV/Morocco/21/KY661857/2008
TOSV/Morocco/XSJT/KM494974/2008
TOSV/Morocco/27SJT/KY661859/2008
TOSV/Morocco/53AJ/KM494972/2011
TOSV/Morocco/24SJT/KY661858/2008
TOSV/Morocco/46AJ/KM494971/2011
TOSV/Tunisia/24/AB904923/2012
TOSV/Tunisia/26/AB904924/2012
TOSV/Tunisia/22/AB904922/2012
TOSV/Morocco/SLP008_10/Morocco/2008/JN832571/2008-07
TOSV/Morocco/28SJT/KY661860/2008
TOSV/France/H4906/KU922127/2004
TOSV/France/H/IMTSSA/FJ153281/2004-07-09
TOSV/France/P233/KU204977/2009
TOSV/France/TOSV_54441132704/KX010934/2015
TOSV/France/9028/KU904265/2007
TOSV/France/11368/KU925899/2009
TOSV/France/TOSV/P51/KU204980/2009
TOSV/France/5963930705/KU935735/2004-2015
TOSV/France/AK/DQ656070/NA
TOSV/France/MRS2010-4319501/KC776216/2010-06-28
TOSV/France/AR/EF656363/2005-07
TOSV/Switzerland/1000414117/MK422498/2018-09-20
TOSV/France/Nice/113/KU204983/2013
TOSV/Spain/LCR_515/2017-07-07
TOSV/Turkey/Bil1/17/TR/MK867803/2017-10-11
TOSV/Turkey/Ku2/17/TR/MK867811/2018-01-10
TOSV/Turkey/Ku1/17/TR/MK867810/2017-12-28
TOSV/Turkey/Kek2/17/TR/MK867807/2017-11-01
TOSV/Turkey/Bil3/17/TR/MK867804/2017-10-16
TOSV/Turkey/Duc4/17/TR/MK867813/2018-01-18
TOSV/Turkey/Bil8/17/TR/MK867805/2017-10-22
TOSV/Turkey/Duc5/17/TR/MK867814/2018-01-25
TOSV/Turkey/Duc3/17/TR/MK867815/2017-12-21
TOSV/Turkey/Duc6/17/TR/MK867812/2018-02-01
TOSV/Turkey/N206np/KU248881/2015-09-01
TOSV/Turkey/N13np/KU248879/2015-05-01
TOSV/Turkey/N90np/KU248880/2015-07-01
TOSV/Turkey/Pige7/17/TR/MK867816/2018-02-15
TOSV/Spain/10310/GU065633/1988
TOSV/Spain/ESH_62100/FJ153279/1993-1999
TOSV/Spain/EsPhGR40/FJ153280/2003-2004
TOSV/Spain/4058515/FJ356705/NA
TOSV/Spain/LCR_756/2018-10-29
TOSV/Spain/42389/GU065639/2001
TOSV/Spain/LCR_654/2017-09-20
TOSV/Spain/22752/GU065638/1992
TOSV/Spain/LCR_228/2015-08-15
TOSV/Spain/LCR_853/2019-09-30
TOSV/Spain/LCR_367/2015-08-20
TOSV/Spain/LCR_623/2017-08-29
TOSV/Spain/ESPh_GR79/GU183147/2004-07
TOSV/Spain/ESPh_GR41/GU065646/2004-07
TOSV/Algeria/A189/KP694240/2008-08
TOSV/Tunisia/T152/JX867534/2010-09-21
TOSV/Tunisia/T166/JX867537/2010-09-27
TOSV/Tunisia/94/AB904919/2011
TOSV/Tunisia/81/AB904918/2011
TOSV/Turkey/SVO/MK413756/2017-05-25
TOSV/Cyprus/66NP/KM111519/2013-07-10
TOSV/Cyprus/24NP/KM111518/2013-07-10
TOSV/Cyprus/6NP/KM111517/2013-07-10
TOSV/Turkey/MA6/MK413751/2018-03-14
TOSV/Turkey/OS10/MK413752/2018-03-26
TOSV/France/A1/KC700343/2010-07
TOSV/France/1500590/DQ975233/2019-03-11
TOSV/France/1500590/MT032308/2019-03-11
TOSV/Italy/CC_INMI1/MZ643218/2019
TOSV/NA/Toscana_ISS.Phl.3/NC_006319/NA
TOSV/Italy/BB8/2014/human/KY626662/2014-07
TOSV/France/5904242804/KU935736/2014
TOSV/Italy/181135_15/2013/KU573067/2013
TOSV/Italy/181135_14/KU573064/2013
TOSV/Greece/Genesi-2012-Greece/KJ418710/2012-06-30
TOSV/Croatia/T8_09/GQ403627/2008
B.1
B.2
B
A
C
97
63
47
64
53
63
100
100
0.07

Figure S4: Phylogenetic divergence tree inferred by IQ-TREE 2 of all labeled TOSV L sequences used, including the seven sequences generated in this study (Andalusia, Spain, 2015-2019). The maximum-likelihood tree includes TOSV segments L sequences retrieved from GenBank as July 2022. Sequences with a minimum length of 190 bp were included. Branch support values for the main lineages are ultrafast bootstrap percentages, and the scale bars represent the number of nucleotide substitutions per site. The genotype as well as proposed lineages B.1 and B.2 are indicated on the right of the tree.

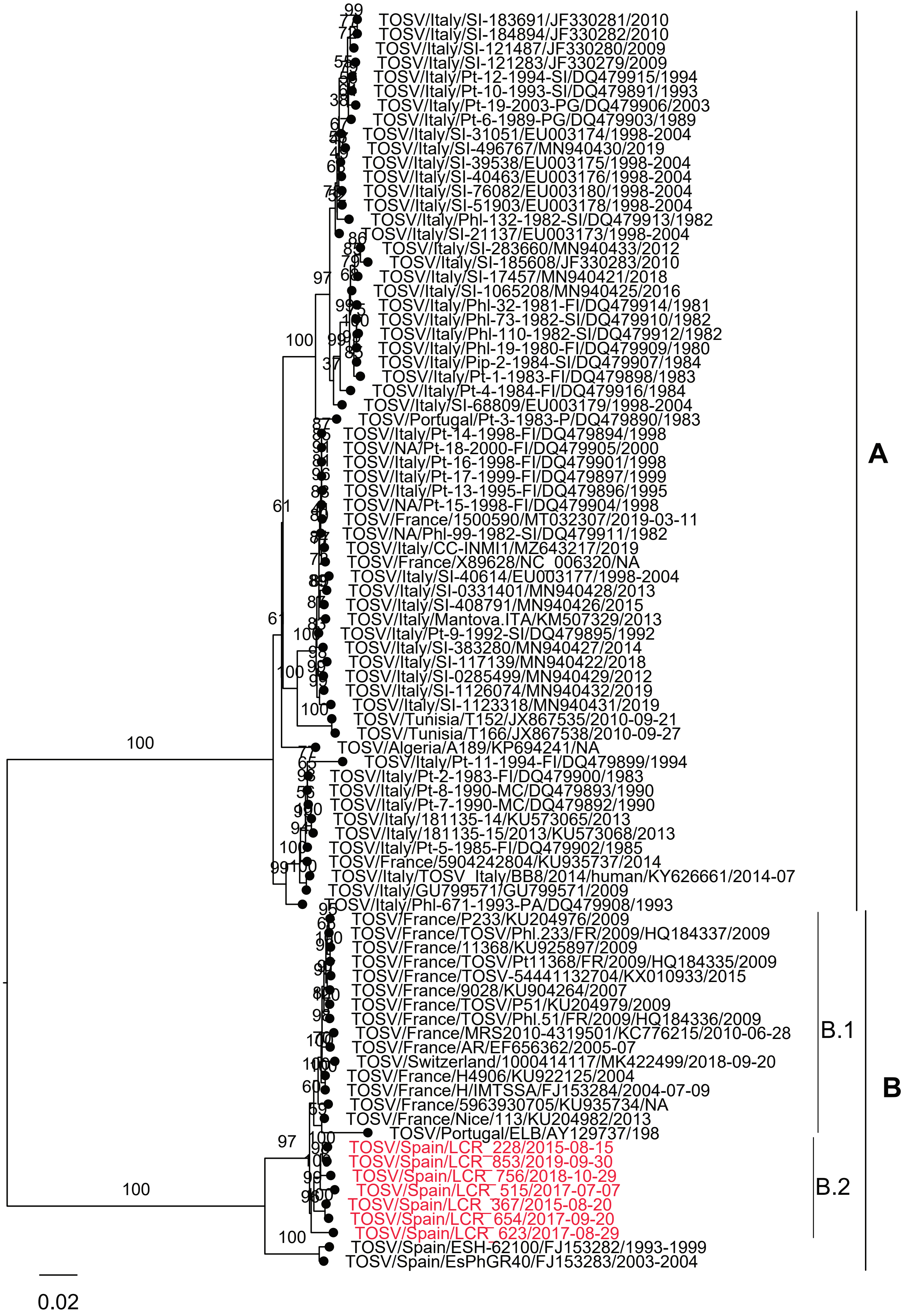
TOSV/Italy/SI-183691/JF330281/2010
TOSV/Italy/SI-184894/JF330282/2010
TOSV/Italy/SI-121487/JF330280/2009
TOSV/Italy/SI-121283/JF330279/2009
TOSV/Italy/Pt-12-1994-SI/DQ479915/1994
TOSV/Italy/Pt-10-1993-SI/DQ479891/1993
TOSV/Italy/Pt-19-2003-PG/DQ479906/2003
TOSV/Italy/Pt-6-1989-PG/DQ479903/1989
TOSV/Italy/SI-31051/EU003174/1998-2004
TOSV/Italy/SI-496767/MN940430/2019
TOSV/Italy/SI-39538/EU003175/1998-2004
TOSV/Italy/SI-40463/EU003176/1998-2004
TOSV/Italy/SI-76082/EU003180/1998-2004
TOSV/Italy/SI-51903/EU003178/1998-2004
TOSV/Italy/Phl-132-1982-SI/DQ479913/1982
TOSV/Italy/SI-21137/EU003173/1998-2004
TOSV/Italy/SI-283660/MN940433/2012
TOSV/Italy/SI-185608/JF330283/2010
TOSV/Italy/SI-17457/MN940421/2018
TOSV/Italy/SI-1065208/MN940425/2016
TOSV/Italy/Phl-32-1981-FI/DQ479914/1981
TOSV/Italy/Phl-73-1982-SI/DQ479910/1982
TOSV/Italy/Phl-110-1982-SI/DQ479912/1982
TOSV/Italy/Phl-19-1980-FI/DQ479909/1980
TOSV/Italy/Pip-2-1984-SI/DQ479907/1984
TOSV/Italy/Pt-1-1983-FI/DQ479898/1983
TOSV/Italy/Pt-4-1984-FI/DQ479916/1984
TOSV/Italy/SI-68809/EU003179/1998-2004
TOSV/Portugal/Pt-3-1983-P/DQ479890/1983
TOSV/Italy/Pt-14-1998-FI/DQ479894/1998
TOSV/NA/Pt-18-2000-FI/DQ479905/2000
TOSV/Italy/Pt-16-1998-FI/DQ479901/1998
TOSV/Italy/Pt-17-1999-FI/DQ479897/1999
TOSV/Italy/Pt-13-1995-FI/DQ479896/1995
TOSV/NA/Pt-15-1998-FI/DQ479904/1998
TOSV/France/1500590/MT032307/2019-03-11
TOSV/NA/Phl-99-1982-SI/DQ479911/1982
TOSV/Italy/CC-INMI1/MZ643217/2019
TOSV/France/X89628/NC_006320/NA
TOSV/Italy/SI-40614/EU003177/1998-2004
TOSV/Italy/SI-0331401/MN940428/2013
TOSV/Italy/SI-408791/MN940426/2015
TOSV/Italy/Mantova.ITA/KM507329/2013
TOSV/Italy/Pt-9-1992-SI/DQ479895/1992
TOSV/Italy/SI-383280/MN940427/2014
TOSV/Italy/SI-117139/MN940422/2018
TOSV/Italy/SI-0285499/MN940429/2012
TOSV/Italy/SI-1126074/MN940432/2019
TOSV/Italy/SI-1123318/MN940431/2019
TOSV/Tunisia/T152/JX867535/2010-09-21
TOSV/Tunisia/T166/JX867538/2010-09-27
TOSV/Algeria/A189/KP694241/NA
TOSV/Italy/Pt-11-1994-FI/DQ479899/1994
TOSV/Italy/Pt-2-1983-FI/DQ479900/1983
TOSV/Italy/Pt-8-1990-MC/DQ479893/1990
TOSV/Italy/Pt-7-1990-MC/DQ479892/1990
TOSV/Italy/181135-14/KU573065/2013
TOSV/Italy/181135-15/2013/KU573068/2013
TOSV/Italy/Pt-5-1985-FI/DQ479902/1985
TOSV/France/5904242804/KU935737/2014
TOSV/Italy/TOSV_Italy/BB8/2014/human/KY626661/2014-07
TOSV/Italy/GU799571/GU799571/2009
TOSV/Italy/Phl-671-1993-PA/DQ479908/1993
TOSV/France/P233/KU204976/2009
TOSV/France/TOSV/Phl.233/FR/2009/HQ184337/2009
TOSV/France/11368/KU925897/2009
TOSV/France/TOSV/Pt11368/FR/2009/HQ184335/2009
TOSV/France/TOSV-54441132704/KX010933/2015
TOSV/France/9028/KU904264/2007
TOSV/France/TOSV/P51/KU204979/2009
TOSV/France/TOSV/Phl.51/FR/2009/HQ184336/2009
TOSV/France/MRS2010-4319501/KC776215/2010-06-28
TOSV/France/AR/EF656362/2005-07
TOSV/Switzerland/1000414117/MK422499/2018-09-20
TOSV/France/H4906/KU922125/2004
TOSV/France/H/IMTSSA/FJ153284/2004-07-09
TOSV/France/5963930705/KU935734/NA
TOSV/France/Nice/113/KU204982/2013
TOSV/Portugal/ELB/AY129737/198
TOSV/Spain/LCR_228/2015-08-15
TOSV/Spain/LCR_853/2019-09-30
TOSV/Spain/LCR_756/2018-10-29
TOSV/Spain/LCR_515/2017-07-07
TOSV/Spain/LCR_367/2015-08-20
TOSV/Spain/LCR_654/2017-09-20
TOSV/Spain/LCR_623/2017-08-29
TOSV/Spain/ESH-62100/FJ153282/1993-1999
TOSV/Spain/EsPhGR40/FJ153283/2003-2004
A
B
B.1
B.2
0.02

Figure S5: Phylogenetic divergence tree inferred by IQ-TREE 2 of all labeled TOSV M sequences used, including the seven sequences generated in this study (Andalusia, Spain, 2015-2019). The maximum-likelihood tree includes TOSV segments M sequences retrieved from GenBank as July 2022. Sequences with a minimum length of 190 bp were included. Branch support values for the main lineages are ultrafast bootstrap percentages, and the scale bars represent the number of nucleotide substitutions per site. The genotype as well as proposed lineages B.1 and B.2 are indicated on the right of the tree.

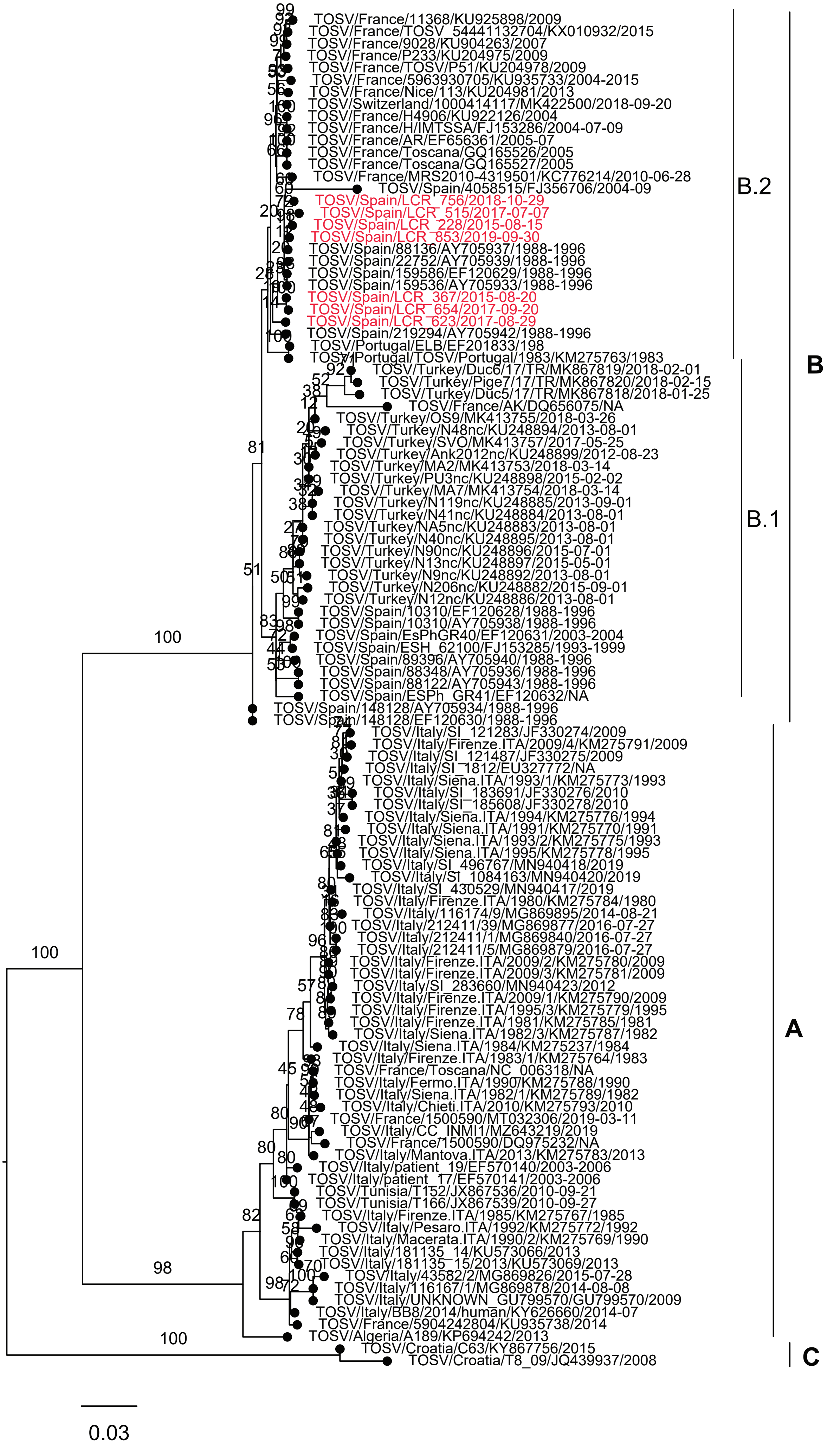
TOSV/France/11368/KU925898/2009
TOSV/France/TOSV_54441132704/KX010932/2015
TOSV/France/9028/KU904263/2007
TOSV/France/P233/KU204975/2009
TOSV/France/TOSV/P51/KU204978/2009
TOSV/France/5963930705/KU935733/2004-2015
TOSV/France/Nice/113/KU204981/2013
TOSV/Switzerland/1000414117/MK422500/2018-09-20
TOSV/France/H4906/KU922126/2004
TOSV/France/H/IMTSSA/FJ153286/2004-07-09
TOSV/France/AR/EF656361/2005-07
TOSV/France/Toscana/GQ165526/2005
TOSV/France/Toscana/GQ165527/2005
TOSV/France/MRS2010-4319501/KC776214/2010-06-28
TOSV/Spain/4058515/FJ356706/2004-09
TOSV/Spain/LCR_756/2018-10-29
TOSV/Spain/LCR_515/2017-07-07
TOSV/Spain/LCR_228/2015-08-15
TOSV/Spain/LCR_853/2019-09-30
TOSV/Spain/88136/AY705937/1988-1996
TOSV/Spain/22752/AY705939/1988-1996
TOSV/Spain/159586/EF120629/1988-1996
TOSV/Spain/159536/AY705933/1988-1996
TOSV/Spain/LCR_367/2015-08-20
TOSV/Spain/LCR_654/2017-09-20
TOSV/Spain/LCR_623/2017-08-29
TOSV/Spain/219294/AY705942/1988-1996
TOSV/Portugal/ELB/EF201833/198
TOSV/Portugal/TOSV/Portugal/1983/KM275763/1983
TOSV/Turkey/Duc6/17/TR/MK867819/2018-02-01
TOSV/Turkey/Pige7/17/TR/MK867820/2018-02-15
TOSV/Turkey/Duc5/17/TR/MK867818/2018-01-25
TOSV/France/AK/DQ656075/NA
TOSV/Turkey/OS9/MK413755/2018-03-26
TOSV/Turkey/N48nc/KU248894/2013-08-01
TOSV/Turkey/SVO/MK413757/2017-05-25
TOSV/Turkey/Ank2012nc/KU248899/2012-08-23
TOSV/Turkey/MA2/MK413753/2018-03-14
TOSV/Turkey/PU3nc/KU248898/2015-02-02
TOSV/Turkey/MA7/MK413754/2018-03-14
TOSV/Turkey/N119nc/KU248885/2013-09-01
TOSV/Turkey/N41nc/KU248884/2013-08-01
TOSV/Turkey/NA5nc/KU248883/2013-08-01
TOSV/Turkey/N40nc/KU248895/2013-08-01
TOSV/Turkey/N90nc/KU248896/2015-07-01
TOSV/Turkey/N13nc/KU248897/2015-05-01
TOSV/Turkey/N9nc/KU248892/2013-08-01
TOSV/Turkey/N206nc/KU248882/2015-09-01
TOSV/Turkey/N12nc/KU248886/2013-08-01
TOSV/Spain/10310/EF120628/1988-1996
TOSV/Spain/10310/AY705938/1988-1996
TOSV/Spain/EsPhGR40/EF120631/2003-2004
TOSV/Spain/ESH_62100/FJ153285/1993-1999
TOSV/Spain/89396/AY705940/1988-1996
TOSV/Spain/88348/AY705936/1988-1996
TOSV/Spain/88122/AY705943/1988-1996
TOSV/Spain/ESPh_GR41/EF120632/NA
TOSV/Spain/148128/AY705934/1988-1996
TOSV/Spain/148128/EF120630/1988-1996
TOSV/Italy/SI_121283/JF330274/2009
TOSV/Italy/Firenze.ITA/2009/4/KM275791/2009
TOSV/Italy/SI_121487/JF330275/2009
TOSV/Italy/SI_1812/EU327772/NA
TOSV/Italy/Siena.ITA/1993/1/KM275773/1993
TOSV/Italy/SI_183691/JF330276/2010
TOSV/Italy/SI_185608/JF330278/2010
TOSV/Italy/Siena.ITA/1994/KM275776/1994
TOSV/Italy/Siena.ITA/1991/KM275770/1991
TOSV/Italy/Siena.ITA/1993/2/KM275775/1993
TOSV/Italy/Siena.ITA/1995/KM275778/1995
TOSV/Italy/SI_496767/MN940418/2019
TOSV/Italy/SI_1084163/MN940420/2019
TOSV/Italy/SI_430529/MN940417/2019
TOSV/Italy/Firenze.ITA/1980/KM275784/1980
TOSV/Italy/116174/9/MG869895/2014-08-21
TOSV/Italy/212411/39/MG869877/2016-07-27
TOSV/Italy/212411/1/MG869840/2016-07-27
TOSV/Italy/212411/5/MG869879/2016-07-27
TOSV/Italy/Firenze.ITA/2009/2/KM275780/2009
TOSV/Italy/Firenze.ITA/2009/3/KM275781/2009
TOSV/Italy/SI_283660/MN940423/2012
TOSV/Italy/Firenze.ITA/2009/1/KM275790/2009
TOSV/Italy/Firenze.ITA/1995/3/KM275779/1995
TOSV/Italy/Firenze.ITA/1981/KM275785/1981
TOSV/Italy/Siena.ITA/1982/3/KM275787/1982
TOSV/Italy/Siena.ITA/1984/KM275237/1984
TOSV/Italy/Firenze.ITA/1983/1/KM275764/1983
TOSV/France/Toscana/NC_006318/NA
TOSV/Italy/Fermo.ITA/1990/KM275788/1990
TOSV/Italy/Siena.ITA/1982/1/KM275789/1982
TOSV/Italy/Chieti.ITA/2010/KM275793/2010
TOSV/France/1500590/MT032306/2019-03-11
TOSV/Italy/CC_INMI1/MZ643219/2019
TOSV/France/1500590/DQ975232/NA
TOSV/Italy/Mantova.ITA/2013/KM275783/2013
TOSV/Italy/patient_19/EF570140/2003-2006
TOSV/Italy/patient_17/EF570141/2003-2006
TOSV/Tunisia/T152/JX867536/2010-09-21
TOSV/Tunisia/T166/JX867539/2010-09-27
TOSV/Italy/Firenze.ITA/1985/KM275767/1985
TOSV/Italy/Pesaro.ITA/1992/KM275772/1992
TOSV/Italy/Macerata.ITA/1990/2/KM275769/1990
TOSV/Italy/181135_14/KU573066/2013
TOSV/Italy/181135_15/2013/KU573069/2013
TOSV/Italy/43582/2/MG869826/2015-07-28
TOSV/Italy/116167/1/MG869878/2014-08-08
TOSV/Italy/UNKNOWN_GU799570/GU799570/2009
TOSV/Italy/BB8/2014/human/KY626660/2014-07
TOSV/France/5904242804/KU935738/2014
TOSV/Algeria/A189/KP694242/2013
TOSV/Croatia/C63/KY867756/2015
TOSV/Croatia/T8_09/JQ439937/2008
B.2
B.1
B
A
C
0.03

Figure S6: Phylogenetic divergence tree inferred by IQ-TREE 2 of all labeled TOSV S sequences used, including the seven sequences generated in this study (Andalusia, Spain, 2015-2019). The maximum-likelihood tree includes TOSV segments M sequences retrieved from GenBank as July 2022. Sequences with a minimum length of 190 bp were included. Branch support values for the main lineages are ultrafast bootstrap percentages, and the scale bars represent the number of nucleotide substitutions per site. The genotype as well as proposed lineages B.1 and B.2 are indicated on the right of the tree.

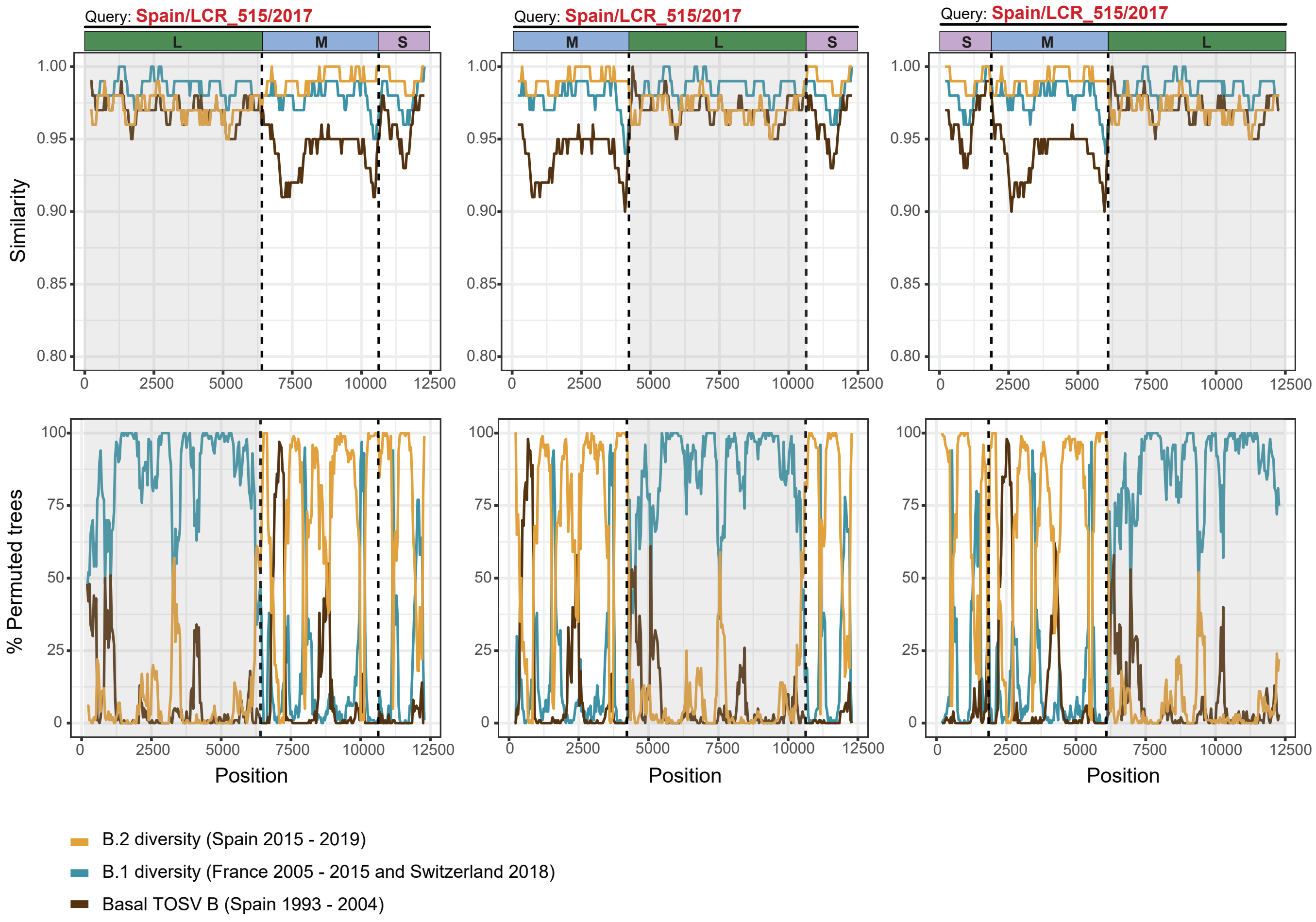

Query: Spain/LCR_515/2017
L
M
S
Similarity
% Permuted trees
Position
B.2 diversity (Spain 2015 - 2019)
B.1 diversity (France 2005 - 2015 and Switzerland 2018)
Basal TOSV B (Spain 1993 - 2004)

Figure S7: Recombination analysis of concatenated TOSV segments analyzing the sequence TOSV/Spain_LCR_515/2017 (Andalusia, Sapin, 2017) with sequences from the B.1 (cyan) and B.2 (yellow) diversity (n=21 total sequences in the analysis). At the top is the similarity plot and at the bottom is the bootscanning analysis plot. The order in which the three TOSV segments were concatenated is shown in the top panel. Analyses were conducted by using SimPlot 3.5.1 (Kimura distance model, window size 400 bp moving in 40 nucleotides steps).

Table S1: Epidemiological, clinical details, and routine laboratory testing performed in all selected samples. Abbreviations: EV: enterovirus; CSF: cerebrospinal fluid; GI: gastrointestinal symptoms; Gluco: glucose; NA: not available; Prot: proteins; WBC: White Blood Cells; HSV: Herpes Simplex Virus; HHV: Human Herpesvirus; H.INF: Haemophilus influenzae; WNV: West Nile Virus; VZV: Varicella Zoster Virus; HIV: human immunodeficiency virus; CMV: Cytomegalovirus; HAV: Hepatitis A Virus; HBV: Hepatitis B Virus; HCV: Hepatitis C virus; EBV: Epstein-Barr virus.

*Living in rural areas refer to individuals living in villages with 900-9000 inhabitants

Sex is aggregated as follows: control positive: 8 females/5 males, control negative: 4 females/4 males; idiopathic: 6 females/17 males

Sample Nº	Living *	Age category	Clinical Diagnosis	Classification	NGS result	Clinical features	Referred insect bites?
28	Urban	51-60	Cognitive impairment	control neg			
559	Rural	71-80	Cognitive impairment	control neg			
695	Rural	61-70	Cognitive impairment	control neg			
901	Rural	31-40	Epilepsy	control neg			
912	Rural	51-60	Cognitive impairment	control neg			
919	Urban	51-60	Epilepsy	control neg			
928	Rural	21-30	Epilepsy	control neg			
673	Urban	91-100	Epilepsy	control neg		Fever, epilepsy	
53	Urban	31-40	Aseptic Meningitis caused by Enterovirus	control pos	Echovirus 13	Fever, headache, nausea, GI	
138	Urban	11-20	Aseptic Meningitis caused by Enterovirus	control pos	Echovirus 6	Fever, headache, vomiting, neck stiffness, GI	
255	Rural	31-40	Aseptic Meningitis caused by Enterovirus	control pos	Echovirus 6	Fever, headache, nausea, GI	

265	Rural	21-30	Aseptic Meningitis caused by Enterovirus	control pos	Echovirus 30	Fever, headache, neck stiffness	
268	Urban	21-30	Aseptic Meningitis caused by Enterovirus	control pos	Echovirus 6	Fever, headache, vomiting, nausea	
365	Rural	21-30	Aseptic Meningitis caused by Enterovirus	control pos	Echovirus 6	Fever, headache	
484	Urban	21-30	Aseptic Meningitis caused by Enterovirus	control pos	Echovirus 11	Fever, headache, nausea	
519	Rural	31-40	Aseptic Meningitis caused by Enterovirus	control pos	Echovirus 30	Fever, headache, vomiting	
520	Rural	21-30	Aseptic Meningitis caused by Enterovirus	control pos	Echovirus 30	Fever, headache, photophobia, GI	
525	Urban	21-30	Aseptic Meningitis	control pos	Mumps	Headache, vomiting, photophobia, sonophobia	
675	Rural	11-20	Aseptic Meningitis caused by Enterovirus	control pos	Echovirus 30	Fever, headache, photophobia, vomiting	
1059	Urban	21-30	Aseptic Meningitis caused by Enterovirus	control pos	Echovirus 11	Fever, headache, photophobia, GI	
1106	Urban	31-40	Aseptic Meningitis caused by Enterovirus	control pos	Echovirus 11	Fever, headache	
228	Rural	41-50	Aseptic Meningitis	idiopath ic	Toscana Virus	Fever, headache, vomiting, nausea, GI, maculopapular rash	Patient referred explicitly no insect bites
367	Urban	11-20	Aseptic Meningitis	idiopath ic	Toscana Virus	Fever, headache, vomiting, nausea, GI, neck stiffness, photophobia, sonophobia	not referred in the clinical record
515	Urban	11-20	Aseptic Meningitis	idiopath ic	Toscana Virus	Fever, headache, vomiting, GI, neck stiffness	Patient referred explicitly no insect bites
623	Urban	21-30	Aseptic Meningitis	idiopath ic	Toscana Virus	Headache, photophobia, sonophobia,	Patient referred explicitly no insect bites
654	Urban	31-40	Aseptic Meningitis	idiopath ic	Toscana Virus	Fever, headache, neck stiffness, photophobia	Patient referred insect bites in a recent travel to Portugal
756	Urban	41-50	Aseptic Meningitis	idiopath ic	Toscana Virus	Fever, headache, vomiting, neck stiffness	Patient referred explicitly no insect bites
853	Rural	71-80	Aseptic Meningoencephalitis	idiopath ic	Toscana Virus	Fever, mental confusion	Patient referred explicitly no insect bites
1152	Rural	51-60	Aseptic Meningoencephalitis	idiopath ic	Toscana Virus	Headache, vomiting. Cattle Farm worker	Patient referred explicitly no insect bites in last days. He remember a tick bite one year ago
59	Urban	81-90	Aseptic Meningitis	idiopath ic		Fever, neck stiffness, mental confusion	not referred in the clinical record
90	Urban	71-80	Aseptic Meningitis	idiopath ic		Fever, vomiting, GI, mental confusion	not referred in the clinical record
165	Urban	31-40	Aseptic Meningitis	idiopath ic		Headache, seizures	not referred in the clinical record
218	Urban	31-40	Aseptic Meningoencephalitis	idiopath ic		Fever, headache, vomiting, neck stiffness, mental confusion	not referred in the clinical record
256	Rural	21-30	Aseptic Meningitis	idiopath ic		Fever, headache, neck stiffness	not referred in the clinical record
437	Rural	41-50	Aseptic Meningitis	idiopath ic		Fever, headache, neck stiffness	Patient referred explicitly no insect bites
553	Rural	11-20	Aseptic Meningoencephalitis	idiopath ic		Fever, headache, vomiting, neck stiffness, sonophobia	Patient referred insect bite one week before
645	Urban	61-70	Aseptic Meningitis	idiopath ic		Mental confusion, language disorder	not referred in the clinical record
649	Urban	41-50	Aseptic Meningitis	idiopath ic		Fever, headache, vomiting	Patient referred explicitly no insect bites
1002	Urban	21-30	Aseptic Meningitis	idiopath ic		Headache, vomiting, nausea, photophobia	not referred in the clinical record
1034	Urban	21-30	Aseptic Meningitis	idiopath ic		Fever, headache, vomiting, GI, neck stiffness	not referred in the clinical record
1047	Urban	31-40	Aseptic Meningitis	idiopath ic		Fever, headache, vomiting	Patient referred explicitly no insect bites

1060	Rural	41-50	Aseptic Meningoencephalitis	idiopathic		Headache, vomiting, nausea	not referred in the clinical record
1114	Urban	41-50	Aseptic Meningoencephalitis	idiopathic		Headache, language disorder	not referred in the clinical record
1150	Urban	61-70	Aseptic Meningoencephalitis	idiopathic		Dysarthria, language disorder, somnolence	not referred in the clinical record

**Sample Nº**	**Month sample collection**	**Year sample collection**	**Length of Hospital Stay (days)**	**CSF analysis**				
				**RBC mm3**	**WBC mm3**	**Lymph %**	**Gluco (mg/dl)**	**Prot (mg/dl)**
28	Feb	2016	1	300	3	NA	80	64
559	Oct	2017	1	100	9	NA	61	26
695	Mar	2018	10	400	1	NA	57	28
901	Feb	2019	4	0	6	NA	58	37
912	Jun	2019	1	300	4	NA	61	52
919	Feb	2019	8	0	0	NA	87	41
928	Oct	2019	13	100	1	NA	53	19
673	Jan	2018	12	1	0	0	134	41
53	May	2016	10	2000	812	72	50	113
138	May	2015	2	700	300	48	94	41
255	Apr	2015	4	100	233	42	59	69
265	Feb	2016	1	100	101	90	53	45
268	Apr	2015	6	100	118	63	55	46
365	May	2015	2	100	203	61	63	41
484	Nov	2015	2	NA	86	90	53	47
519	Mar	2018	2	300	422	91	NA	63
520	May	2018	4	100	310	82	NA	41
525	Apr	2018	3	200	747	95	57	54
675	Mar	2017	3	0	156	63	60	58
1059	Dic	2018	3	383	383	72	NA	68
1106	Dic	2018	2	NA	312	90	55	63
228	Aug	2015	6	0	15	87	46	96
367	Aug	2015	2	100	154	94	59	62
515	Jul	2017	10	200	97	97	59	48
623	Ago	2017	3	200	1710	63	38	61
654	Sep	2017	2	200	246	82	60	135
756	Nov	2018	4	0	138	96	54	74
853	Oct	2019	14	100	235	90	41	106
1152	Aug	2019	16	0	273	95	158	109
59	Feb	2016	18	100	40	87	77	59
90	Oct	2016	5	200	157	91	52	74
165	Sep	2015	6	500	119	83	58	64
218	Dec	2015	15	100	300	99	56	133
256	Nov	2015	14	11700	191	68	45	63
437	Aug	2016	1	4800	72	90	70	38
553	Nov	2017	3	200	228	100	74	87
645	Apr	2018	6	500	31	97	74	70

649	Jul	2018	8	200	928	95	48	110
1002	May	2018	3	0	71	71	79	47
1034	Apr	2019	9	NA	79	100	NA	NA
1047	Jul	2016	3	300	137	87	56	68
1060	Oct	2017	16	300	311	94	37	118
1114	Apr	2018	10	540000	404	79	66	109
1150	Jun	2019	4	300	258	98	11	121

**Sample Nº**	**Routine Clinical Laboratory tests performed during hospitalization**				
	**PCRs EVs and HHV-1,2,3,4,5,6**	**PCR Bacterial Meningitis (N. MEN, S. PNEU, LISTERIA, E.COLI, H.INF TIPO B)**	**Culture CSF**	**Serology COXIELLA, BORRELIA, RICKETSIA, BRUCELLA**	**Others**
28					
559					Serology Treponema pallidum
695					
901					
912					
919					
928					
673					
53	YES	YES			
138	YES				
255	YES	YES	YES		
265	YES		YES		
268	YES				
365	YES				
484	YES	YES			
519	YES	YES			
520	YES				
525					PCR Mumps
675	YES	YES	YES	YES	
1059	YES	YES	YES	YES	Serology VZV, Parvovirus, Mycoplasma pneumoniae
1106	YES	YES	YES		
228	YES	YES	YES	YES	Serology Treponema pallidum
367	YES	YES	YES		
515	YES	YES	YES		
623	YES	YES	YES	YES	Serology HIV, VZV
654	YES	YES	YES		
756	YES	YES	YES	YES	Serology VZV, Parvovirus, Treponema pallidum, Mycoplasma pneumoniae
853	YES	YES	YES		Serology HIV, VZV, CMV, Treponema pallidum
1152	YES	YES	YES	YES	PCR Toscana virus, WNV. PCR and culture micobacterias, Serology Treponema pallidum, CMV, EBV, HIV, HBV, HCV. PCR Cryptococcus
59	YES	YES	YES	YES	PCR Micobacterias, Serology Treponema pallidum, Serology HIV
90	YES	YES			

165	YES	YES	YES		Serology Treponema pallidum
218	YES	YES	YES		PCR Micobacterias
256	YES	YES	YES		
437	YES	YES	YES	YES	Serology HIV, VZV, HBV, HAV, HCV, Treponema pallidum
553	YES		YES		Cryptococcus Antigen in CSF
645	YES	YES	YES		Serology HIV, VZV
649	YES	YES	YES	YES	Serology HIV, VZV, Mycoplasma pneumoniae
1002	YES				
1034	YES	YES	YES		Serology HIV, HBV, CMV
1047	YES	YES	YES	YES	
1060	YES	YES	YES	YES	Cryptococcus Antigen in CSF, Serology Influenza A, B, Mycoplasma pneumoniae, PCR Borrelia in CSF
1114	YES	YES	YES	YES	Serology HIV, VZV, EBV, Treponema pallidum, PCR CMV in serum
1150	YES	YES	YES		PCR and culture micobacterias, PCR Cryptococcus

Table S2: Description of the laboratory tests applied to the samples in this study.

**Pathogen**	**Type of test**	**Test**
Enterovirus	PCR	Genexpert Xpert EV (Cepheid)
Herpesvirus 1,2,3		Real Cycler Herpesvirus tipo 1 (HSV-1) + Herpesvirus tipo 2 (HSV-2) + Virus Varicela-Zóster (VZV). Ref: HSVTVA-T. (Progenie)
Herpesvirus 4,5,6		Real Cycler Herpesvirus tipo 4 (EBV) + Herpesvirus tipo 5 (CMV) + Herpesvirus tipo 6 (HSV-6). Ref: HERPLxG. (Progenie)
N. meningitidis, S. pneumoniae y L. monocytogenes		Real Cycler Ref. MENELI (Progenie)
Haemophilus Influenziae		FilmArray ME (biomerieux)
Mycobacterium tuberculosis		Genexpert MTB (Cepheid)
Toscana		Perez-Ruiz et al. J. Clin. Virol. 2007 DOI 10.1016/j.jcv.2007.05.003
Treponema pallidum	Serology	Screening with Advia Centaur Syphilis Assay (Siemens). If undetermined or positive is repeated with RPR Carbon test (Chromatest) and TPHA test (Spinreact).
Coxiella		ELISA Coxiella IgM, Coxiella IgG (Vircell)
Rickettsia		ELISA Rickettsia IgM, Rickettsia IgG (Vircell)
Brucella		ELISA Brucella IgG (Euroinmunne)
Borrelia		Chemiluminescence immunoassay (CLIA) Borrelia IgM, Borrelia IgG (Diasorin)
HIV		ADVIA Centaur® HIV Ag/Ab Combo (CHIV) Assay (Siemens). Confirmatory test with INNO-LIA® HIV I/II Score (Fujirebio)
Mycoplasma pneumoniae		ELISA IgM and IgG (Vircell)
Cryptococcus		Filmarray ME (Biomerieux)
Hepatitis virus A, B and C		ADVIA Centaur HCV, HAV assays, ADVIA Centaur HBsAg assay (Siemens)

Parvovirus		ELISA IgM and IgG (Euroinmunne)
Varicella Zoster Virus		Chemiluminescence immunoassay (CLIA) Liaison® VZV IgM and IgG (Diasorin)
Epstein-Barr virus		Chemiluminescence immunoassay (CLIA) Liaison® EBV IgM and IgG (Diasorin)
Cytomegalovirus		Chemiluminescence immunoassay (CLIA) Liaison® CMV IgM and IgG (Diasorin)
Bacterial screening	Culture	Chocolate agar, blood agar and thioglycollate broth

Table S3: Taxa names and metadata of complete TOSV genomes (n=29).

					Accession Numbers			
**Name**	**Strain**	**Country**	**Date**	**Nextstrain date**	**L**	**M**	**S**	**Host**
TOSV/France/AR/2005-07	AR	France	2005-07		EF656363	EF656362	EF656361	Sandfly
TOSV/Spain/EsPhGR40/2003-2004	EsPhGR40	Spain	2003-2004	2003-June	FJ153280	FJ153283	EF120631	Sandfly
TOSV/France/H/IMTSSA/2004-07-09	H/IMTSSA	France	2004-07-09		FJ153281	FJ153284	AY766034	Human
TOSV/Tunisia/T152/2010-09-21	T152	Tunisia	2010-09-21		JX867534	JX867535	JX867536	Sandfly
TOSV/France/MRS2010-4319501/2010-06-28	MRS2010-4319501	France	2010-06-28		KC776216	KC776215	KC776214	Human
TOSV/Algeria/A1	A189	Algeria	2013-08		KP694240	KP694241	KP694242	Sandfly

89/2013-08								
TOSV/France/P233/2009	P233	France	2009		KU204977	KU204976	KU204975	Sandfly
TOSV/France/TOSV/P51/2009	P51	France	2009		KU204980	KU204979	KU204978	Sandfly
TOSV/France/Nice/113/2013	Nice/113	France	2013		KU204983	KU204982	KU204981	Sandfly
TOSV/Italy/181135-14/2013	181135-14	Italy	2013		KU573064	KU573065	KU573066	Sandfly
TOSV/Italy/181135-15/2013	181135-15	Italy	2013		KU573067	KU573068	KU573069	Sandfly
TOSV/France/9028/2007	9028	France	2007		KU904265	KU904264	KU904263	Human
TOSV/France/H4906/2004	H4906	France	2004		KU922127	KU922125	KU922126	Human
TOSV/France/11368/2009	11368	France	2009		KU925899	KU925897	KU925898	Human
TOSV/France/5963930705/2004-2015	5963930705	France	2004-2015	2010	KU935735	KU935734	KU935733	Human
TOSV/France/5904242804/2014	5904242804	France	2014		KU935736	KU935737	KU935738	Human
TOSV/France/TO	TOSV_54441132704	France	2015		KX010934	KX010933	KX010932	Human

SV_5444 1132704 /2015								
TOSV/Italy/BB8/2014/human/2014-07	TOSV_Italy/BB8/2014/human	Italy	2014-07		KY626662	KY626661	KY626660	Human
TOSV/Spain/LCR_228/2015-08-15	LCR_228	Spain	2015-08-15					Human
TOSV/Spain/LCR_367/2015-08-20	LCR_367	Spain	2015-08-20					Human
TOSV/Spain/LCR_515/2017-07-07	LCR_515	Spain	2017-07-07					Human
TOSV/Spain/LCR_623/2017-08-29	LCR_623	Spain	2017-08-29					Human
TOSV/Spain/LCR_654/2017-09-20	LCR_654	Spain	2017-09-20					Human
TOSV/Spain/LCR_756/2018-10-29	LCR_756	Spain	2018-10-29					Human
TOSV/Spain/LCR_853/2019-09-30	LCR_853	Spain	2019-09-30					Human
TOSV/Switzerland/1000414117/2018-09-20	1000414117	Switzerland	2018-09-20		MK422498	MK422499	MK422500	Human
TOSV/Spain/ESH_	ESH_62100	Spain	1993-1999	1996	FJ153279	FJ153282	FJ153285	Human

62100/1993-1999								
TOSV/Italy/CC_INMI1/2019	CC_INMI1	Italy	2019		MZ643218	MZ643217	MZ643219	Human
TOSV/Tunisia/T166/2010-09-27	T166	Tunisia	2010-09-27		JX867537	JX867538	JX867539	Sandfly

Table S4: TOSV mNGS results from CSF samples.

SAMPLE	SEGMENT	TOTAL NB OF READS	AVERAGE COVERAGE	MAPPED READS (%)	GENOME COVERED (%)
LCR_228	L	5,270,466	34.2	0.0569	98.3
	M		36.8	0.0404	93.3
	S		21.8	0.0107	81.1
LCR_367	L	21,978,596	19.9	0.0079	64.7
	M		5.9	0.0015	29.4
	S		19.6	0.0023	71.9
LCR_515	L	11,158,116	23.5	0.0185	96.5
	M		20.9	0.0109	90.6
	S		10.2	0.0024	66.5
LCR_623	L		62.6	0.0419	99.0

	M	13,147,500	42.6	0.0189	94.4
	S		61.7	0.0121	91.9
LCR_654	L	9,122,218	4.8	0.0047	38.4
	M		NA	0.0030	39.6
	S		9.9	0.0028	65.5
LCR_756	L	11,329,328	5.1	0.0039	38.8
	M		1.6	0.0008	9.6
	S		9.9	0.0022	63.3
LCR_853	L	17,472,260	201.4	0.1014	98.1
	M		118.4	0.0393	96.8
	S		274.2	0.0405	96.7
LCR_1152	L	10,369,600	3.5	0.0030	18.3
	M		0.1	0.0000	0.0
	S		5.3	0.0013	37.9

Table S5: Primers and primer's concentration used in the optimized primer pool scheme.

**Segment**	**Primer**	**Sequence (5' -> 3')**	**pool**	**Final concentration pool (µM)**
L	TOSV_L_1_LEFT	AGCATTAACCATTCATCCCCTGAG	1	0.015
L	TOSV_L_1_RIGHTb	GGTCAGCTGARTTGGCATCACC	1	0.015
L	TOSV_L_3_LEFTb	ATTGCTGTGCATTTTAATGGGGT	1	0.015
L	TOSV_L_3_RIGHTb	AACCTCTCTTTGTTGGTTATATTCTCA	1	0.015

L	TOSV_L_5_LEFTb	CCCTGGGTGACAARAACTGG	1	0.015
L	TOSV_L_5_RIGHTb	CCTGGCCTCTTTGACAGCTC	1	0.015
L	TOSV_L_7_LEFTb	TACTGGCTATGGGYTTGCATCA	1	0.015
L	TOSV_L_7_RIGHTb	ARACAAAGTCGGTCACCAGGAG	1	0.015
L	TOSV_L_9_LEFT	ATCAGGCAAGTGCAGCAAAGTT	1	0.015
L	TOSV_L_9_RIGHT	AAGTAGCCATTGTAGCAGCAGC	1	0.015
L	TOSV_L_11_LEFT	GCCCTCCTACTGACAAGTACCT	1	0.015
L	TOSV_L_11_RIGHTb	TGCTGCTGCTTTTTGAAYAAGC	1	0.015
L	TOSV_L_13_LEFT	GTGCAACCCTACTAACAAAATCAGGA	1	0.015
L	TOSV_L_13_RIGHTb	TGTGCACCCTTGGGAWATCCAT	1	0.015
L	TOSV_L_15_LEFT	TGCAAGGGATTCTACACTTCACC	1	0.015
L	TOSV_L_15_RIGHTb	CCACCTCAGGAAGTGAGCAG	1	0.015
L	TOSV_L_17_LEFT	TGTCAGTGCTCAATACATTACATGCT	1	0.015
L	TOSV_L_17_RIGHTb	CCTCTTGAATTTCTCCACCGACC	1	0.015
L	TOSV_L_19_LEFTb	TAGCAGAGAAGGTGCATAGCC	1	0.015
L	TOSV_L_19_RIGHT	CCTCTGRCTTGCCCTGGGAATA	1	0.015
L	TOSV_L_21_LEFT	GGTTTGGCACCAGAAAGAGTAAGA	1	0.015
L	TOSV_L_21_RIGHTb	CTCTARCTTGGCTTCTTCAGAGTACGG	1	0.015
L	TOSV_L_23_LEFT	CCAACATAATCAGCCTACTGCAGA	1	0.015
L	TOSV_L_23_RIGHTb	GTTGGCTTTTGARACATCCATGTTG	1	0.015
L	TOSV_L_25_LEFTb	GGCCTGTTRCATGATGATGACA	1	0.015
L	TOSV_L_25_RIGHT	TGAGGATTCTGTGCAAAGCCTG	1	0.015
L	TOSV_L_27_LEFT	TGACATGATGYTAGATGAAGGAATGGA	1	0.015
L	TOSV_L_27_RIGHT	GTCGTCAGGAACTTCAGTTGGG	1	0.015
M	TOSVM_2_LEFT	GTTGCTGAGGTGCACTCTAACC	1	0.015
M	TOSVM_2_RIGHT	CTAGCCCTCTCCTTTGCTGTCT	1	0.015
M	TOSVM_4_LEFT	GCCTGATTGCAAAAGAGAAAGCA	1	0.015
M	TOSVM_4_RIGHT	TCTCCCACAGGCATTYGATGAA	1	0.015

M	TOSVM_6_LEFT	CCCCAGTGCAAGATGTRTTCTG	1	0.015
M	TOSVM_6_RIGHT	TCACATGGATACCTGYTGCAGA	1	0.015
M	TOSVM_8_LEFT	GCTGATTGTCAAGACTCATGGTCC	1	0.015
M	TOSVM_8_RIGHT	CACCCAGCAGCAAAARACTGAT	1	0.015
M	TOSVM_10_LEFT	AGCAGACTCCAAGATCATGCAR	1	0.015
M	TOSVM_10_RIGHT	TCAAAGCATCTGTTCTCGTGCA	1	0.015
M	TOSVM_12_LEFT	TGACTCAGARGGRATAACAGGAACA	1	0.015
M	TOSVM_12_RIGHT	CTAATGTGAGGCGGATGGATGC	1	0.015
M	TOSVM_14_LEFT	CGTTCTCCATTTCTCAAGKCCT	1	0.015
M	TOSVM_14_RIGHT	CATACACATGCACTGCCYACTY	1	0.015
S	TOSV_S_1_LEFT	CCTCCCGTATYGCTAAACCAGA	1	0.015
S	TOSV_S_1_RIGHT	GATTCCTGTTGGCCATCTRAGG	1	0.015
S	TOSV_S_3_LEFT	AGGGTTCTCCTAGAAGGYAAGA	1	0.015
S	TOSV_S_3_RIGHT	GACTAGGCAGCCACTTTRTCAC	1	0.015
S	TOSV_S_5_LEFT	TGAGTAGCTTCTTTTTGTCCTCCC	1	0.015
S	TOSV_S_5_RIGHT	AATCCTGGCAGAGACACCATCA	1	0.015
L	TOSV_L_2_LEFTb	GGAGAACAGGGCTGATGAYTCA	2	0.015
L	TOSV_L_2_RIGHT	TCCAGTCTGCTGACAGTCTCA	2	0.015
L	TOSV_L_4_LEFT	AGAGGTTGATGAAGAATACATATCCAAGA	2	0.015
L	TOSV_L_4_RIGHTb	ACCTTATTCCAGATCTTACACATGG	2	0.015
L	TOSV_L_6_LEFT	ACAGAGCGGTCAGTTGAAAGAA	2	0.015
L	TOSV_L_6_RIGHTb	ACTGAGGCAGACAACTCAGC	2	0.015
L	TOSV_L_8_LEFTb	TGATYAAGCCCACAACATCTAAGGG	2	0.015
L	TOSV_L_8_RIGHT	TCTGGGAGCGATACAAACCCTT	2	0.015
L	TOSV_L_10_LEFT	ATGCAGAGGATYTCAGCCACTC	2	0.03
L	TOSV_L_10_RIGHT	TTGGCTCATCACTCCTCTTCCA	2	0.03
L	TOSV_L_12_LEFT	ACTACACAAGGGATAAGCTTCTGGT	2	0.015

L	TOSV_L_12_RIGHTb	GTGTGAACTCGCAGAGCATCA	2	0.015
L	TOSV_L_14_LEFTb	GAGGCTGCTCAATGTTCACYAA	2	0.03
L	TOSV_L_14_RIGHT	TGCTATCATCTGAGCCCTGCAT	2	0.03
L	TOSV_L_16_LEFTb	AGTCCACTCCYAACACCGACTT	2	0.015
L	TOSV_L_16_RIGHTb	AAYCCTGATAGACCAGCAGAGT	2	0.015
L	TOSV_L_18_LEFTb	GGAGGRTCACAGAAAACAGATGG	2	0.015
L	TOSV_L_18_RIGHTb	CTGATGATGCCATGACCTTGCA	2	0.015
L	TOSV_L_20_LEFTb	GTTCGAGGCATTCGACTCAATCATC	2	0.015
L	TOSV_L_20_RIGHTb	TTRCGAATCTGGATGTGGTTGG	2	0.015
L	TOSV_L_22_LEFT	GCAAATCAGGATTCTTGCAAGGG	2	0.03
L	TOSV_L_22_RIGHTb	ATCCATCACCCCCTTCCAGATR	2	0.03
L	TOSV_L_24_LEFTb	GGAAGGAAGGGATGCCATCYCA	2	0.015
L	TOSV_L_24_RIGHT	TGTGCTTTGAACTGACAGAGCT	2	0.015
L	TOSV_L_26_LEFTb	TACAAAGGGGCCTTYTCTGTCG	2	0.03
L	TOSV_L_26_RIGHT	CCTTGTAATGTGCTGGCCCAAA	2	0.03
L	TOSVM_1b_LEFT	GCAAAGTTGTTATTAATTTCCTGTTTAGTGC	2	0.015
L	TOSVM_1b_RIGHT	TGACATCATTGAGGATCTCAACCC	2	0.015
M	TOSVM_3b_LEFT	CCATCCTCYAARAAACATGTTCCG	2	0.015
M	TOSVM_3b_RIGHT	GCCATGAGYAGAGTGGTGAT	2	0.015
M	TOSVM_5b_RIGHT	CTTGCATTCYTTCACGGTGTG	2	0.015
M	TOSVM_5b_LEFT	GTACAGCATGYTAGAAGCTGC	2	0.015
M	TOSVM_7_LEFT	GGCTCATCTGTCAAGCTAAAGAAGT	2	0.015
M	TOSVM_7_RIGHT	GGTACACAAAGGTWGCTGGCTT	2	0.015
M	TOSVM_9_LEFT	CTCTTGCTTGTGAAAGGGGCAA	2	0.015
M	TOSVM_9_RIGHT	GAGCCTATGGGTCCCAATCTCA	2	0.015
M	TOSVM_11_LEFT	GAATGTGTCTCTGATCGHTGCC	2	0.015
M	TOSVM_11_RIGHT	CGAGGCTCTGGAGAATATGGGT	2	0.015
M	TOSVM_13_LEFT	AGGTTCGGGATGGGATGACYTT	2	0.015

M	TOSVM_13_RIGHT	CATCATGTGGAGCAGTGGCAAT	2	0.015
M	TOSVM_15_LEFT	GCATGAAGGAGGTTCTTCTTTAGTRGT	2	0.015
M	TOSVM_15_RIGHT	TGTCCAAACTGCCATCAACTTGT	2	0.015
S	TOSV_S_2_LEFT	GAGCCAACAATGAGGGARCTTT	2	0.015
S	TOSV_S_2_RIGHT	CAATTTAGTCACCCGYGCTGCT	2	0.015
S	TOSV_S_4_LEFT	GCTCTTTCCACCTTYTGGGCTA	2	0.09
S	TOSV_S_4_RIGHT	TAAGCAGGCTAATGAGGTGGCT	2	0.09
S	TOSV_S_6_LEFT	CAGAAACTGGCAGYGATTCTGA	2	0.015
S	TOSV_S_6_RIGHT	CGCGACATTGCTCTTGCTTTTC	2	0.015

Table S6: TOSV amplicon-based sequencing results from CSF samples.

SAMPLE	SEGMENT	TOTAL NB OF READS	AVERAGE COVERAGE	MAPPED READS (%)	GENOME COVERED (%)
LCR_228	L	26,036,074	104,289	59.6	92.7
	M		82,122	30.9	93.4
	S		49,627	8.3	93.1
LCR_367	L	14,332,700	39,732	42.2	78.7
	M		57,434	40.2	79.1
	S		40,549	12.6	93.1
LCR_515	L	13,504,654	51,314	57.7	96.4
	M		43,734	32.4	97
	S		21,050	6.9	93.1
LCR_623	L	10,416,918	36,578	53.4	96.4
	M		33,657	32.4	97

	S		23,004	9.8	93.1
LCR_654	L	15,046,180	60,901	61.6	90.4
	M		34,548	23	92.1
	S		34,514	10.2	93.1
LCR_756	L	5,841,460	21,491	56	94.8
	M		16,182	27.8	97
	S		16,692	12.7	93.1
LCR_853	L	5,234,954	18,406	53.4	96.4
	M		18,570	35.5	97
	S		9,257	7.9	93.1
